# Epidemiological analysis of infectious diseases in older people in China from 2014 to 2022: a population-based study

**DOI:** 10.1016/j.lanwpc.2025.101729

**Published:** 2025-11-03

**Authors:** Sheng-Hong Lin, Chen-Long Lv, Meng-Jie Geng, Rui-Cheng Gao, Yan-Qun Sun, Yan-He Wang, Ya-Ming Zheng, Tian Tang, Chun-Xi Shan, Yao Tian, Yun-Bo Qiu, Jun Ma, Yan Zhang, Yu-Feng Yang, Qiang Xu, Guo-Lin Wang, Yan-Ping Zhang, Qun Li, Li-Ping Wang, Peng-Tao Bao, Li-Qun Fang, Wei Liu

**Affiliations:** a State Key Laboratory of Pathogen and Biosecurity, Academy of Military Medical Science, Beijing, China; b Division of Infectious Disease Control and Prevention, National Key Laboratory of Intelligent Tracking and Forecasting for Infectious Diseases, Chinese Center for Disease Control and Prevention, Beijing, China; c Department of Urology, Urology Research Institute, The First Affiliated Hospital, Fujian Medical University, Fuzhou, Fujian, China; d Clinical Research Center, Children's Hospital of Nanjing Medical University, Nanjing, China; e The 968th Hospital of Joint Logistics Support Force of PLA, Jinzhou, Liaoning, China; f Chinese Center for Disease Control and Prevention, Beijing, China; g Senior Department of Pulmonary and Critical Care Medicine, The Eighth Medical Center of Chinese PLA General Hospital, Beijing, China

**Keywords:** Infectious diseases, Public health, Older people, National surveillance

## Abstract

**Background:**

The whole world is undergoing an unprecedented rapid population aging, and the accompanying threat of infectious diseases epidemic among the older people will pose a serious challenge to the national public health.

**Methods:**

We extracted individual data on 21 notifiable infectious diseases (NIDs) among individuals aged ≥60 years from the Chinese Infectious Disease Surveillance and Control Project (CISDCP) from 2014 to 2022 in the mainland of China. We comprehensively analyzed the epidemiological characteristics of NIDs in older people and estimated the impact of age, year, and the COVID-19 pandemic.

**Findings:**

A total of 8,604,064 cases of 21 major NIDs were reported, with an overall annual incidence of 362·10/100,000. Sexually transmitted or bloodborne diseases (STBDs, 45·23%) were the most common diseases. Notably, ranking of syphilis rose from fourth to third, while HIV/AIDS moved from eighth to seventh; respiratory diseases (34·88%) showed sustained declines. Tuberculosis remained the most common respiratory diseases despite a 6·4% annual decline (APC, P < 0·001); gastrointestinal or enteroviral diseases (GEDs, 18·35%) showed sustained declines, except for infectious diarrhea and hepatitis E; vector-borne or zoonotic diseases (VBZDs, 1·54%) remained low, though brucellosis increased during COVID-19 pandemic. Inflection point analysis revealed that 13 diseases, such as tuberculosis, infectious diarrhea, hepatitis B, and others, exhibited a quadratic distribution (inverted “V” shape) in age-specific incidence as age increased. Difference was noticeable between regions, with the older people in Inner Mongolia-Xinjiang region and South China region continuing to carry a disproportionate burden from NIDs.

**Interpretation:**

Although China's success in infectious disease control in older people. Effective prevention and control strategies are needed for highest incidence diseases, such as tuberculosis, hepatitis B, and infectious diarrhea, especially in high-incidence regions and among critical age points.

**Funding:**

National Major Research & Development Program of China (2022YFC2604000).

**Research in context**

**Evidence before this study**

We searched PubMed on April 20, 2025, with the terms (“older people” OR “aging”) AND (“infectious diseases” OR “tuberculosis” OR “hepatitis B” OR “infectious diarrhea” OR “hepatitis C” OR “syphilis” OR “seasonal influenza”) for articles published in English. Our search did not identify any reports of recent trends and epidemiological features of infectious diseases in the older people in China. We retrieved a paper on the incidence characteristics of infectious diseases in the older people in Shandong Province, China, and we found one study analyzed the spatial spillover effect of environmental factors on the incidence of tuberculosis among the older people; and 21 studies analyzed the risk factors for the incidence of infectious diseases such as tuberculosis, seasonal influenza, AIDS, and COVID-19 among the older people. To date, no study has yet systematically investigated the recent epidemic trends of infectious diseases among the older people population in large-population developing countries, including China, within the broader context of global population aging.

**Added value of this study**

Based on the national surveillance data of notifiable infectious diseases (NIDs) from 2014 to 2022, our study was the first to comprehensively analyzed the epidemiological characteristics of NIDs in the older people and estimated the impact of age, year, and the COVID-19 pandemic, since China entered an aging society (the proportion of the population aged ≥60 years accounts for 10% or more of the total population in a given country or region) in 1999. Currently, the main category of infectious diseases threatening the health of the older people was the STBDs. Apart from STBDs, the incidences of all the other categories exhibited a decreasing trend. STBDs such as hepatitis B, syphilis, hepatitis C, and HIV/AIDS exhibited a rising pattern in the annual incidence among the older people, underscoring the need for continued prioritization of STBDs in public health strategies. A significant disparity in disease incidence was observed across six age groups, and the highest incidences were observed among 60–64 years group for STBDs and VBZDs, among 75–79 years group for respiratory diseases, among 80–84 years group for GEDs. Notably, a higher incidence was observed among males than females, and in rural than urban areas. VBZDs had the highest incidence in small cities, while the other three disease categories had the highest incidence in super cities. Most NIDs exhibit a quadratic distribution (13/18) trend in the age-specific incidence with increasing age; however, it should be noted that seasonal influenza and bacterial dysentery demonstrate a linear increase pattern in incidence with age. The older people witnessed a significant decline in GEDs and VBZDs during the initial stage of the COVID-19 pandemic, with reductions of 35·74% and 20·88% in incidence, respectively.

**Implications of all the available evidence**

To identify the incidence of infectious diseases among the older people in China, and to investigate their spatial and temporal characteristics, as well as the effect from age, year, and COVID-19 pandemic within the context of global population aging, which is crucial for effectively reducing the disease burden in the aging-population era. STBDs such as hepatitis B, syphilis, hepatitis C, and HIV/AIDS have increased in incidence prior to COVID-19 and require continued attention. Significant disparities exist in terms of sex, urban versus rural residence, and geographic regions, necessitating the development of targeted and region-specific public health strategies to effectively address the health challenges faced by the older people.

## Introduction

The global trend of population aging, driven by longer life expectancy and reduced fertility rates, has significantly shaped the social population structure and disease pattern in the 21st century, posing significant challenges to public health systems worldwide.^1^ China, as the most populous developing country, is experiencing an unprecedented acceleration in its aging population.^2^ According to the United Nations World Population Prospects 2022, China's older people aged 60 and above is projected to rise to 36·5% by 2050.^3^ The health challenge faced by the older people constitute a large portion of the overall public health burden across all age groups. Data from the World Health Organization's 2018 World Health Statistics reveal that while the average life expectancy in China is 76·4 years; the disease-free life span is only 68·7 years. This implies that older people spend approximately eight years living with illnesses,^4^ a phenomenon highlighting the critical issue of “longevity without healthy life” among the older people.^5^ Notably, this age group faces a significantly higher risk of notifiable infectious diseases (NIDs) between 2004 and 2013, with incidence and case-fatality rates approximately three times higher than in non-older people.^6^

NIDs are those infectious diseases that are clearly stipulated by national laws and regulations and must be reported to the health administrative authorities once diagnosed. Currently, China's NIDs are classified into three categories, including Category A, B, and C, comprising a total of 41 diseases.^7^ The increased susceptibility to NIDs and diminished vaccine efficacy in the older people can be partially attributed to age-related immune system changes.^8^ For instance in Shandong Province, tuberculosis exhibits a significantly higher incidence among the older people compared to younger demographics, remaining as the leading cause of mortality among respiratory infectious diseases in China.^9^^,^^10^ Additionally, in recent years, the mortality burden associated with seasonal influenza has been substantial and increasing among older people.^10^^,^^11^ Notably, from 2004 to 2018 in China, the age-specific mortality rate for ten respiratory infectious diseases (e.g., tuberculosis, epidemic cerebrospinal meningitis, seasonal influenza) was highest among individuals aged 60 or older.^10^

This study aims to comprehensively analyze the epidemiological characteristics of major NIDs in the older people and assess the impact of age, year, and the COVID-19 pandemic using national surveillance data from 2014 to 2022. By doing so, it seeks to deepen the understanding of NIDs epidemiological patterns in the older people, which is crucial for effectively reducing the disease burden in the aging-population era.

## Methods

### Study population and data collection

World Health Organization defines older people as those aged ≥60 years in developing countries, such as China, and as individuals ≥65 years of age in developed countries.^1^^,^^5^^,^^12^ In this study, we adopted China's threshold age of 60 years for defining the older people.^13^ We retrieved individual-level data of all reported NIDs cases from the Chinese Infectious Disease Surveillance and Control Project (CISDCP) for the period 2014–2022. The CISDCP is an Internet-based real-time disease-reporting system that covers 41 NIDs. This system has expanded to encompass 55,077 national health facilities across 397 cities in all 31 provinces in the mainland of China. According to data from China's seventh national population census, the total population of all 31 provinces in the mainland of China is 1411·78 million. Among them, the number of people aged 60 and above is 264·02 million. In line with the guidelines issued by the National Health Commission of the People's Republic of China, standardized individual case data are electronically transmitted from grassroots institutions within 24 h of clinical or laboratory diagnosis.^14^^,^^15^ Over the past decade, the CISDCP database used in our study has maintained a national average coverage rate ranging from 85·1% to 95·4%. In 2017, it covered 87·2% of medical institutions nationwide. The coverage rate was higher at the county level and above (95·8%) than at the township level (85·5%). Regionally, the coverage rates were 95·1% in the eastern regions, 94·5% in the central regions, and 81·6% in the western regions.^15^ All NIDs were diagnosed according to their standard diagnostic criteria. Data uploaded by hospitals will be further verified for the laboratory test results, and checked for errors and duplications. In this study, viral hepatitis is further categorized into hepatitis A, B, C, D, and E, as well as untyped, each distinguished by specific transmission routes, clinical manifestations, and associated viral species. Similarly, dysentery is divided into bacterial dysentery and amoebic dysentery. In total, 46 kinds of NIDs were reported in the CISDCP. We first quantified the relative disease burden among older people, then excluded cases under the age of 60, and included diseases for which older people reported more than 100 cases per year during the study period. Hepatitis (untyped) and COVID-19 were excluded from the analysis. Eliminate ineligible data such as duplicate cases, indeterminate cases, and outliers (Appendix pp 2 and 16). Finally, 21 NIDs were remained and categorized into four groups: respiratory diseases (encompassing three diseases), gastrointestinal or enteroviral diseases (GEDs, eight diseases), sexually transmitted or bloodborne diseases (STBDs, five diseases), and vector-borne or zoonotic diseases (VBZDs, five diseases; Appendix p 5). For each reported case, we extracted demographic information (including age, sex, occupation, and residential address code), diagnosis information, outcome data (recovered or succumbed), as well as the timeline of symptom onset, diagnosis, and outcome determination.

The core indicator is the incidence, defined as the ratio of the number of new cases of infectious disease in a given population during a specified time period to the number of individuals who were susceptible during the same period (per 100,000). Population estimates were derived by aggregating data on the older people from national, and provincial and prefecture-level statistics that were estimated regarding the calendar year, age group, and sex from 2014 to 2022, using data from the National Bureau of Statistics (NBS) and China Population and Employment Statistical Yearbook.

### Statistical analysis

We first quantified the relative disease burden among the older people by calculating the incidence rate ratio (IRR) between the older people and the younger adults. On this basis, descriptive analyses were conducted to examine the trends in incidences and the case number for the 21 NIDs in the older people from 2014 to 2022. Age and province specific disease in each survey year and the seasonal variation of each disease by month were analyzed. To identify the features of major infectious diseases we extracted the top infectious disease for each age and ten-year age intervals. To analyze the epidemiological characteristics of NIDs in different subgroups of the older people, we stratified the data by gender into male and female groups. For age groups, we divided the population into the following categories: 60–64 years, 65–69 years, 70–74 years, 75–79 years, 80–84 years, and 85 years and above. Regarding place of residence, we categorized the cases into rural and urban areas. Urban areas were further classified based on population size into super city, megacity, large city, medium-sized city, and small city. Additionally, we divided the cases into seven ecological regions: Northeast region, North China region, Inner Mongolia-Xinjiang region, Qinghai-Tibet region, Southwest region, Central China region, and South China region. We also categorized the cases by occupation into farmers, retired individuals, domestic/unemployed, and others. Frequencies and percentages were calculated for categorical variables, and comparison between groups was conducted using Pearson's Chi-square test or Fisher's exact test, as appropriate. P < 0·05 was considered statistically significant. All tests were two-sided.

Chronic infectious diseases are a category of infections characterized by prolonged duration, slow progression, and resistance to rapid cure. These conditions are typically caused by viruses, bacteria, or other pathogens that the immune system cannot fully eliminate. Acute infectious diseases, in contrast, are infections with short duration and rapid progression, usually reaching peak severity within a short period. These diseases are commonly caused by bacteria, viruses, parasites, or fungi and can be transmitted directly or indirectly between individuals (Appendix p 5).^16^^,^^17^ These 21 NIDs were classified into 14 acute NIDs, such as mumps, seasonal influenza, hepatitis A, hepatitis E, hand, foot and mouth disease (HFMD), typhoid and paratyphoid, bacterial dysentery, acute hemorrhagic conjunctivitis, infectious diarrhea, amoebic dysentery, gonorrhea, typhus, hemorrhagic fever with renal syndrome (HFRS), and rabies, as well as seven chronic NIDs, including tuberculosis, syphilis, HIV/AIDS, hepatitis B, hepatitis C, brucellosis, and hydatid disease. This classification was based on their duration and progression rate.

The Join-Point regression (JPR) model was utilized to analyze the temporal trend and potential flection points in annual incidence over time, as well as age-specific incidences across various age groups for each NIDs. The study period for the age-specific incidence is the average annual incidence from 2014 to 2022, with age grouped in one-year increments (e.g., one year, two years, ... up to 100 years). The JPR model allows for the evaluation of statistically significant changes (flection points) in trends. When statistically significant year(s) or age group(s) were determined, linear regression techniques were employed to estimate the regression parameters.

The JPR model for the observations (*x*_*1*_, *y*_*1*_), …, (*x*_*n*_, *y*_*n*_), where *x*_*1*_ ≤ ... ≤*x*_*n*_ without loss of generality, can be written as:

$$E\left[\log\left(y\right)|x\right]=\beta_{0}+\beta_{1}x+\delta_{1}{\left(x-\tau_{1}\right)}^{+}+\ldots +\delta_{k}{\left(x-\tau_{k}\right)}^{+}$$

where *y* is the outcome of interest (annual incidence or age-specific incidence), *x* is the calendar year or age group series, the *τ*_*k*_ ‘s are the unknown join-points and α^+^ = α for α > 0 (α = x-τ_j_, k is the total number of connections and j is the index (j = 1, 2, …, k), representing the jth connection) and 0 otherwise.^18^ Linear trends were estimated as the annual percentage change (APC) using a natural log-linear model, calculated as APC_i_ = [(Exp (*β*_*i*_)−1)] × 100, where *β*_*i*_ represents the slope of the period segment. A positive APC value signifies an upward trend, while a negative APC value indicates a downward trend.^19^ In our analysis, we applied the JPR model to the log-transformed incidence rates to better capture the trends and potential inflection points over time. The log transformation is commonly used in epidemiological studies to stabilize variance and make the data more normally distributed, which is essential for accurate trend analysis. This analysis was conducted using the JPR Program, version 4·9·1·0 (Statistical Research and Applications Branch, National Cancer Institute, USA).

To investigate the impact of Nonpharmaceutical interventions (NPIs) against COVID-19 on the epidemic of NIDs, the nine-year study period was divided into three distinct phases according to the temporal dynamics of the pandemic: pre-COVID-19 pandemic (January 1, 2014–January 22, 2020), COVID-19 pandemic Stage I (January 23, 2020–April 30, 2020), and COVID-19 pandemic Stage II (May 1, 2020–November 30, 2022). We compared the incidences of each NIDs during Stage I and Stage II of the COVID-19 pandemic with the average incidences in the corresponding time periods during the pre-COVID-19 pandemic. For each disease, the percentage of change (PC) in incidence was calculated as following:

$$\frac{i_{{time}_{1}}\left(p\right)-i_{{time}_{2}}\left(p\right)}{i_{{time}_{2}}\left(p\right)}\;\times \;100\%$$

where ${time}_{1}$ (Stage I: January 23, 2020–April 30, 2020; Stage II: May 1, 2020–November 30, 2022) refers to either Stage I or Stage II of the COVID-19 pandemic, while ${time}_{2}$ (January 1, 2014–January 22, 2020) indicates the pre-COVID-19 pandemic. $i_{{time}_{1}}\left(p\right)$ represent the incidence or average incidence during phase *p* within ${time}_{1}$, and $i_{{time}_{2}}\left(p\right)$ represents the incidence or average incidence specific to phase *p* within ${time}_{2}$. All statistical analyses were conducted using R software (version 4·3·2; Appendix p 3).

### Ethics approval

The study was approved by the Institutional Review Board of the Academy of Military Medical Science (IRB number: AF/SC-08/02.343). Written informed consent was waived by the National Health Commission of China for the surveillance of NIDs. All the data of cases used in this study were anonymized and personal identification information is not included in the data.

### Role of the funding source

The funder of the study had no role in the study design, data collection, data analysis, data interpretation, or writing of the report. The corresponding authors had full access to all the data and had final responsibility for the decision to submit for publication.

## Results

### Incidence of NIDs in older people versus younger adults

Overall, a higher burden of NIDs was observed in older people, with an incidence rate ratio (IRR) of 1·170 (95% CI: 1·169, 1·171; P < 0·001) compared to younger adults (18‒59-year-olds; Appendix pp 6 and 7). Diseases that were significantly more incidence in older people than in younger adults included tuberculosis (IRR = 1·760; 95% CI: 1·757, 1·763; P < 0·001), infectious diarrhea (IRR = 1·439; 95% CI: 1·436, 1·442; P < 0·001), hepatitis C (IRR = 1·488; 95% CI: 1·483, 1·493; P < 0·001), and rabies (IRR = 2·803; 95% CI: 2·625, 2·993; P < 0·001), etc. HFMD has the highest incidence in the entire population and among those under 17 years of age. Hepatitis B has the highest incidence among younger adults. Tuberculosis has the highest incidence among older people (Appendix p 17).

### Incidence of four categories of NIDs

**Fig. 1:**
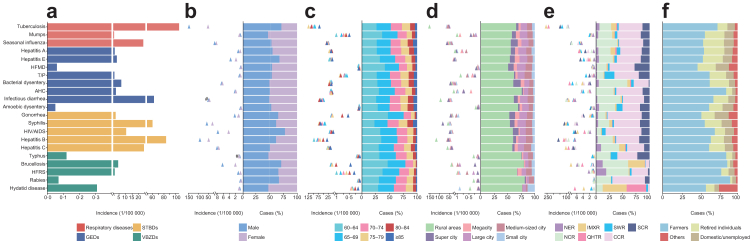
**Incidences and proportions of different infectious diseases among the older people stratified by subgroups.** a. Disease. b. Sex. c. Age group (years). d. Residence. Super city: urban resident population over ten million; Megacity: urban resident population between five million and ten million; Large city: urban resident population between one million and five million; Medium-sized city: urban resident population between 500,000 and 1,000,000 people; Small city: urban resident population less than 500,000. e. Ecological zone. f. Occupation. HFMD: hand, foot and mouth disease; T/P: typhoid and paratyphoid; AHC: acute hemorrhagic conjunctivitis; HFRS: hemorrhagic fever with renal syndrome. GEDs: gastrointestinal or enteroviral diseases; STBDs: sexually transmitted or bloodborne diseases; VBZDs: vector-borne or zoonotic diseases. NER: Northeast region; NCR: North China region; IMXR: Inner Mongolia-Xinjiang region; QHTR: Qinghai-Tibet region; SWR: Southwest region; CCR: Central China region; SCR: South China region. ∗Indicates that 21 diseases are significantly different across age groups, residence and ecological zones. # indicates that there are significant differences between genders for the remaining 19 diseases, except for infectious diarrhea, and typhus.

From 2014 to 2022, a total of 8,604,064 individuals aged ≥60 years were reported with an overall annual incidence of 362·10/100,000 population (Table 1). STBDs, respiratory diseases, GEDs and VBZDs accounted for 45·23%, 34·88%, 18·35% and 1·54% of all reported cases, respectively. Among the 21 NIDs, tuberculosis had the highest incidence, followed by hepatitis B, infectious diarrhea, syphilis, hepatitis C, and seasonal influenza. These six diseases accounted for 90·39% of all cases. Among five of the STBDs, hepatitis B was reported with the highest case number (20·92% of all cases) and annual incidence (75·76 per 100,000), followed by syphilis (annul incidence of 50·80 per 100,000). Among three respiratory diseases, tuberculosis was reported as the highest incidence, with an annual incidence at 101·90/100,000. GEDs constitute of 18·35% of the reported cases, with infectious diarrhea (14·25% of all cases) that indicates a group of infectious diseases with diarrhea as the main symptom caused by various pathogens other than cholera, dysentery, typhoid and paratyphoid, being the predominant disease. GEDs have an annual incidence of 66·39/100,000, with infectious diarrhea reporting the highest annual incidence of 51·58/100,000. Among five VBZDs, brucellosis was reported as the highest incidence, with an annual incidence of 4·22/100,000 (Fig. 1a; Appendix p 8).

**Table 1:** Demographic characteristics of different categories of NIDs among the older people in China, 2014–2022.

Characteristic	Respiratory diseases		GEDs		STBDs		VBZDs	
	Cases^b^ (incidence^c^)	P-value	Cases^b^ (incidence^c^)	P-value	Cases^b^ (incidence^c^)	P-value	Cases^b^ (incidence^c^)	P-value
Sex		P < 0·001		P < 0·001		P < 0·001		P < 0·001
Male	2,027,271 (176·83)		781,430 (68·16)		2,370,298 (206·75)		90,656 (7·91)	
Female	973,822 (79·19)		796,198 (64·75)		1,521,741 (123·75)		42,648 (3·47)	
Age group (years)		P < 0·001		P < 0·001		P < 0·001		P < 0·001
60–64	810,779 (122·76)		425,214 (64·38)		1,203,551 (182·23)		58,956 (8·93)	
65–69	773,139 (116·08)		393,027 (59·01)		1,051,728 (157·91)		41,934 (6·30)	
70–74	589,987 (132·19)		294,742 (66·04)		732,160 (164·05)		19,971 (4·47)	
75–79	431,251 (153·39)		216,587 (77·04)		478,817 (170·31)		8322 (2·96)	
80–84	259,212 (141·30)		150,284 (81·92)		269,554 (146·94)		3079 (1·68)	
≥85	136,725 (98·53)		97,774 (70·46)		156,229 (112·59)		1042 (0·75)	
Residence		P < 0·001		P < 0·001		P < 0·001		P < 0·001
Rural areas	1,907,551 (174·65)		964,067 (88·27)		2,272,426 (208·06)		104,050 (9·53)	
Urban areas	1,093,542 (85·17)		613,561 (47·79)		1,619,613 (126·14)		29,254 (2·28)	
Urban areas^a^		P < 0·001		P < 0·001		P < 0·001		P < 0·001
In super city	174,311 (108·83)		119,510 (74·61)		251,409 (156·96)		653 (0·41)	
In megacity	138,606 (100·70)		72,842 (52·92)		188,198 (136·73)		3932 (2·86)	
In large city	343,090 (72·18)		200,265 (42·13)		498,460 (104·87)		8025 (1·69)	
In medium-sized city	290,434 (80·21)		170,616 (47·12)		470,658 (129·99)		7252 (2·00)	
In small city	147,101 (98·90)		50,328 (33·84)		210,888 (141·78)		9392 (6·31)	
Ecological zone^d^		P < 0·001		P < 0·001		P < 0·001		P < 0·001
Northeast region	147,274 (77·27)		42,008 (22·04)		150,166 (78·78)		16,324 (8·56)	
North China region	747,654 (94·01)		681,191 (85·65)		969,224 (121·87)		59,986 (7·54)	
Inner Mongolia-Xinjiang region	265,761 (294·00)		48,029 (53·13)		232,177 (256·85)		35,163 (38·90)	
Qinghai-Tibet region	32,464 (234·78)		5782 (41·81)		34,763 (251·40)		2809 (20·31)	
Southwest region	87,281 (125·47)		40,438 (58·13)		117,939 (169·54)		2375 (3·41)	
Central China region	1,320,834 (134·15)		593,056 (60·23)		1,729,736 (175·67)		14,091 (1·43)	
South China region	399,825 (172·45)		167,124 (72·08)		658,034 (283·82)		2556 (1·10)	
Total (8,604,064, 362·10/100,000)	3,001,093 (126·30)		1,577,628 (66·39)		3,892,039 (163·80)		133,304 (5·61)	

GEDs, gastrointestinal or enteroviral diseases; STBDs, sexually transmitted or bloodborne diseases; VBZDs, vector-borne or zoonotic diseases.

P-values were calculated using the Pearson chi-square test or Fisher's exact test to assess the statistical significance of differences in incidence between subgroups. P < 0·05 was considered statistically significant. All tests were two-sided.

a Super city: urban resident population over ten million; Megacity: urban resident population between five million and ten million; Large city: urban resident population between one million and five million; Medium-sized city: urban resident population between 500,000 and 1,000,000 people; Small city: urban resident population less than 500,000.

b Total cases for nine years.

c Yearly incidence (per 100,000), average for nine years.

d The distribution of ecological zone was shown in Appendix p 18.

### Regional patterns of major NIDs

**Fig. 2:**
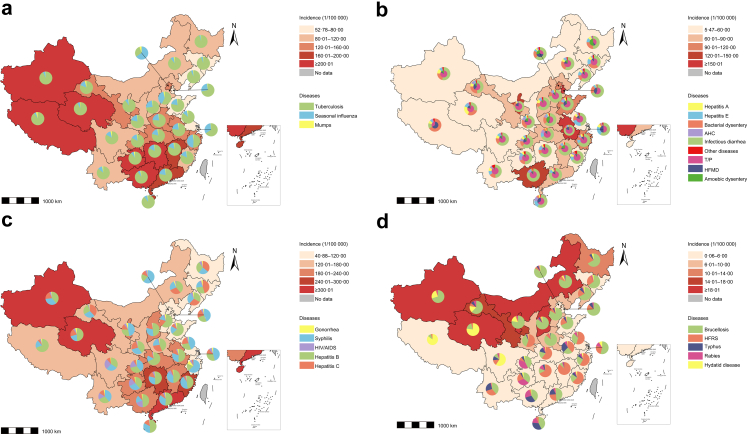
**Geographical distribution of incidences and proportions of different categories of infectious diseases among the older people in China, 2014–2022.** a. Respiratory diseases. b. Gastrointestinal or enteroviral diseases. c. Sexually transmitted or bloodborne diseases. d. Vector-borne or zoonotic diseases. HFMD: hand, foot and mouth disease; T/P: typhoid and paratyphoid; AHC: acute hemorrhagic conjunctivitis; HFRS: hemorrhagic fever with renal syndrome. The large pie charts in Panels b show the distribution of the top five diseases and the “other diseases” category in terms of the number of cases in each province, while the small pie charts further refine the “other diseases” category (which refers to the diseases that ranked low in each province).

The regional distributions of disease patterns varied significantly across four major categories (Table 1; Appendix p 18). For respiratory diseases, annual incidences were significantly higher in Inner Mongolia-Xinjiang region (P < 0·001). Tuberculosis ranked as the highest incidence disease across all provinces/metropolises, except for Beijing, where seasonal influenza was more frequently reported (Fig. 2a). Regarding GEDs, higher incidences were observed in North and South China region. Infectious diarrhea had the highest incidence in all provinces except Tibet, where bacterial dysentery accounted for an exceptionally high proportion (Fig. 2b). For STBDs, the overall incidences were higher in South China region, although variations were observed across different geographic areas. Hepatitis B exhibited higher proportion of cases in central, south, and northwestern China; syphilis had higher proportion of cases in eastern China; hepatitis C had the highest proportion of cases in Gansu, Heilongjiang and Jilin Provinces; HIV/AIDS had the highest proportion of cases in Southwest region China (Fig. 2c). For VBZDs, the overall incidence was higher in Inner Mongolia-Xinjiang region, mainly attributed to the higher proportion of brucellosis in this region. HFRS had the highest proportion of cases in the Central China region (Fig. 2d).

Significantly higher incidences were observed in rural areas compared to urban areas across all four disease categories. Among urban settings, super cities with a resident population exceeding ten million exhibited the highest incidences for three of the disease categories. In contrast, for VBZDs, the highest incidence was observed in small cities with a population of less than 500,000 residents (all P < 0·001) (Table 1). Although 21 NIDs exhibited the highest proportion of cases in rural areas, the highest incidence rates of seasonal influenza, bacterial dysentery, gonorrhea, syphilis, and HIV/AIDS were observed in super cities (Fig. 1d). In addition, significant regional variations in the incidence of NIDs were observed. For instance, tuberculosis, hepatitis C, and brucellosis were most common in the Inner Mongolia-Xinjiang region. Mumps, typhoid and paratyphoid, and HIV/AIDS exhibited the highest incidence in Southwest region, while seasonal influenza, hepatitis B, and syphilis showed higher incidence in the South China region (all P < 0·001). Moreover, Central China region had the highest proportion of cases for the majority of NIDs (Fig. 1e).

Furthermore, most chronic infectious diseases, including respiratory diseases (101·90/100,000), STBDs (161·81/100,000), and VBZDs (4·52/100,000), were observed to exhibit predominantly higher incidences than acute infectious diseases across nearly all provinces. Exceptions include Beijing and several provinces in the central and southern China, where the incidence of acute respiratory diseases (Seasonal influenza) and VBZDs (HFRS, and typhus) was notably higher than that of chronic respiratory diseases and VBZDs, respectively (Appendix p 9).

### Temporal features of major NIDs

**Fig. 3:**
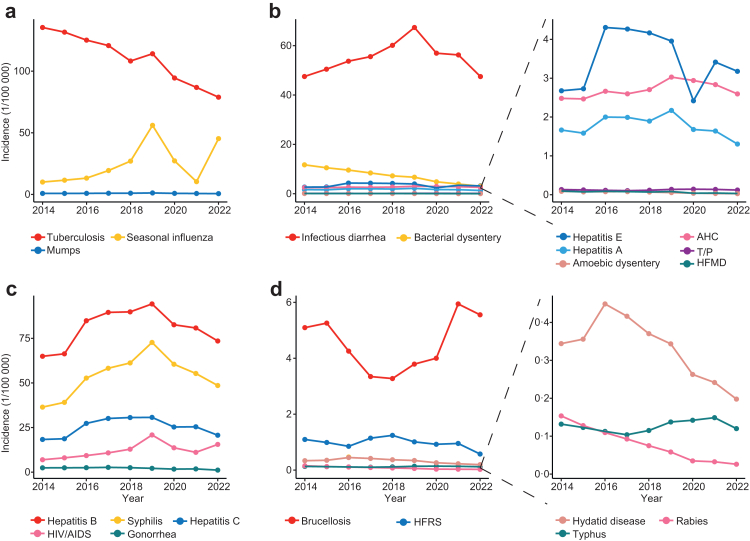
**Temporal trends in the incidences of different categories of NIDs among the older people in China, 2014–2022.** a. Respiratory diseases. b. Gastrointestinal or enteroviral diseases. c. Sexually transmitted or bloodborne diseases. d. Vector-borne or zoonotic diseases. HFMD: hand, foot and mouth disease; T/P: typhoid and paratyphoid; AHC: acute hemorrhagic conjunctivitis; HFRS: hemorrhagic fever with renal syndrome.

**Fig. 4:**
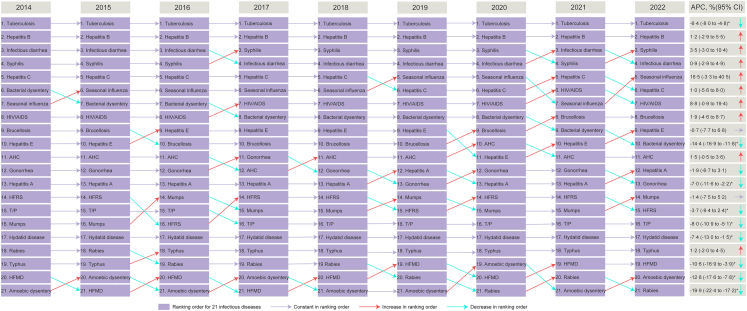
**Ranking of incidences of each of the 21 NIDs by year, from 2014 to 2022.** Annual percentage change (APC) in incidence of each infectious disease is listed with 95% confidence intervals. In the APC column, which corresponds to the same 21 diseases as in 2022, arrows pointing upwards (red) represent overall increased trends, arrows pointing downwards (green) represent decreased trends, whereas arrows pointing to the right (purple) represent stable trends for incidence of each infectious disease from 2014 to 2022. HFMD: hand, foot and mouth disease; T/P: typhoid and paratyphoid; AHC: acute hemorrhagic conjunctivitis; HFRS: hemorrhagic fever with renal syndrome.

From 2014 to 2022, the incidences of all NIDs categories except STBDs showed a downward trend. Conversely, STBDs exhibited an overall rise before the COVID-19 pandemic, followed by a decline during the pandemic (Appendix p 19). Respiratory diseases—Tuberculosis had a significant annual decline, with annual percentage change (APC) of −6·4% (P < 0·001), but the incidence remained the highest. Seasonal influenza, decreasing during the COVID-19 period, rising from seventh in 2014 to fifth in 2022 in incidence rankings of all 21 included NID_S_ (APC 16·5%, P = 0·094). Most GEDs experienced a sustained decline in the incidence in recent years. Bacterial dysentery and amoebic dysentery have experienced APC of 14·4% and 12·8% (both P < 0·001), respectively. However, infectious diarrhea and hepatitis E initially increased, peaking in 2019 and 2016, respectively, before declining. Prior to 2019, four of the five STBDs increased, but this trend reversed after 2019, except for gonorrhea. Ranking of syphilis rose from fourth to third, while HIV/AIDS moved from eighth to seventh. Notably, HIV/AIDS resurged significantly in 2022. Most syphilis cases were latent syphilis (86·65%), increasing from 68·73% to 93·48% over the study period. In contrast, primary syphilis (from 20·86% to 3·68%) declined during this period (Appendix p 10). VBZDs generally had low incidences. Brucellosis increased during the COVID-19 pandemic, peaking in 2021 and rising from ninth to eighth (Figs. 3 and 4). In addition, the incidence of tuberculosis in the entire population also showed a downward trend between 2014 and 2022, while the incidence of seasonal influenza, infectious diarrhea, syphilis, and hepatitis B exhibited an upward trend, with seasonal influenza reaching its peak in 2019. Notably, the incidence of these diseases was relatively lower among people under 17 years of age (Appendix p 20).

Regarding the seasonal patterns of NIDs in older people, tuberculosis and seasonal influenza exhibited peak incidences during winter, particularly in January. Conversely, syphilis cases showed a marked decline in February. The majority of GEDs reached their peak incidence during the summer months (June to August). However, VBZDs exhibited more pronounced seasonal variations. For instance, brucellosis primarily peaked in early summer (May to June, Appendix p 21).

### Demographic features of major NIDs

Across all four disease categories, males consistently exhibited higher incidences compared to females (Table 1). Notably, males had significantly higher incidence for 17 NIDs compared to females, while mumps, and hydatid disease had lower incidences in males than in females (all P < 0·001, Fig. 1b). For age-specific incidence, a disparity in disease incidence was observed across the six pre-defined age groups, with the highest incidence noted among the 60–64 years group for STBDs and VBZDs, among the 75–79 years group for respiratory diseases, and among the 80–84 years group for GEDs (Table 1). Among the 21 individual diseases, 12 exhibited the highest incidence in individuals aged 60–64 years (all P < 0·001). Tuberculosis, hepatitis A, typhoid and paratyphoid, syphilis, and hydatid disease had the highest incidence in individuals aged 75–79 years, while seasonal influenza, bacterial dysentery, infectious diarrhea, and amoebic dysentery had the highest incidence in those aged 80–84 years (all P < 0·001). Additionally, 20 NIDs exhibited the highest proportion of cases in individuals aged 60–64 years, except syphilis (65–69 years, Fig. 1c). Notably, 21 NIDs exhibited the highest proportion of cases among farmers (Fig. 1f).

### Age-specific incidence trends

Among different age groups, hepatitis B, seasonal influenza, and syphilis are the main threats for the older people in 2022 compared to 2014 (Appendix p 22). During the study period, the incidence of tuberculosis was the highest among individuals aged under 88 years every year. However, in the age group of 90–98 years in 2019 and in those aged ≥89 years in 2022, the incidence of seasonal influenza was the highest. Among GEDs, infectious diarrhea has the highest annual incidence among all age groups. Hepatitis B was the STBDs with the highest incidence among people aged under 74 years during 2014–2022. In contrast, syphilis exhibited a distinct pattern over time and across different age groups. In 2014, the highest incidence of syphilis was observed in individuals aged 81 years or older. From 2015 to 2019, this highest incidence progressively shifted to those aged over 75 years. Subsequently, between 2020 and 2022, the highest incidence of syphilis gradually shifted again, this time to individuals aged over 78 years. For VBZDs, brucellosis had the highest incidence during the study period, but HFRS incidence was highest in the aged ≥86 years, especially from 2016 to 2020 (Appendix p 23).

**Fig. 5:**
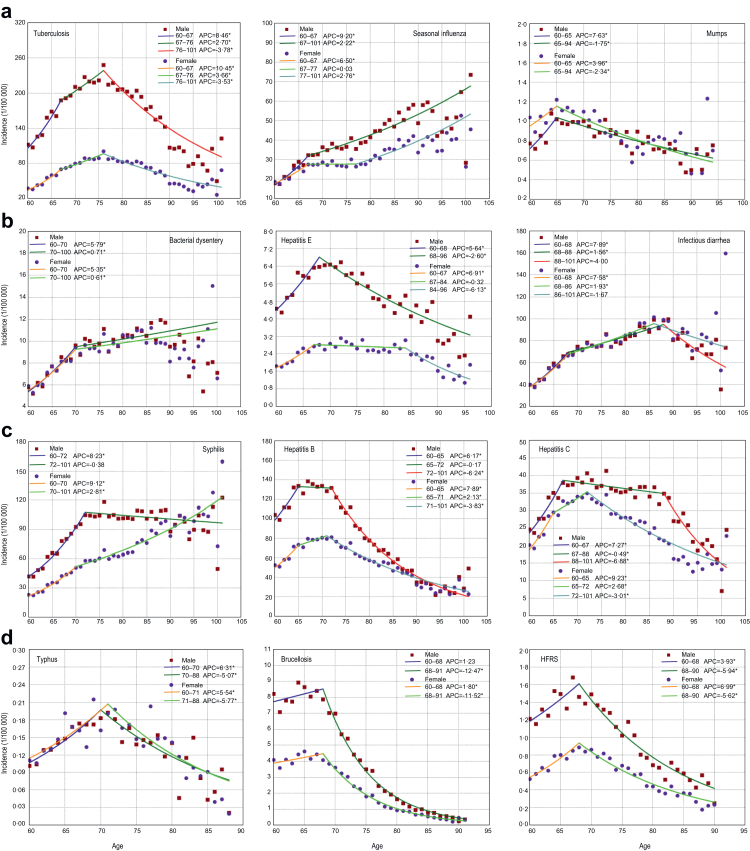
**Age-dependent changing trends in age-specific incidences of different infectious diseases among the older people, stratified by sex as analyzed using Join-Point regression model.** a. Respiratory diseases. b. Gastrointestinal or enteroviral diseases. c. Sexually transmitted or bloodborne diseases. d. Vector-borne or zoonotic diseases. HFRS: hemorrhagic fever with renal syndrome. The points indicate the observed age-specific incidences, and the lines indicate the fitted age-dependent changing trends of incidences. The significant annual percent changes (APCs) at two-sided P < 0·05 are marked by “∗”.

Sex- and location-stratified JPR models revealed distinct trajectories for 18/21 diseases (Fig. 5; Appendix pp 11–13, and 24–30) (excluding HFMD, amoebic dysentery, and rabies due to data discontinuity). Quadratic/declining trends were displayed for tuberculosis, infectious diarrhea, hepatitis B (age-dependent decrease). Sex-dimorphic patterns were observed for the others. For example, syphilis, and typhoid and paratyphoid exhibited a linear increase with age among females, while a quadratic distribution was observed among males; however, it should be noted that seasonal influenza and bacterial dysentery demonstrate a linear increase pattern in incidence with age.

For respiratory diseases, the flection points indicating a decline in incidences of tuberculosis and mumps were observed at 76 and 65 years of age, respectively, for both males and females (both P < 0·001). In urban areas, the inflection points for tuberculosis occurred two years earlier in males compared to females. Notably, no such flection points were observed for seasonal influenza in either sex, as the age-specific incidence of seasonal influenza exhibited a linear increase with advancing age. Furthermore, the inflection points for the decline in tuberculosis incidence in 2022 occurred seven years earlier for males and six years earlier for females compared to 2014.

For GEDs, the inflection points indicating a decline in incidences of hepatitis E and infectious diarrhea appeared one year and two years later in males compared to females, respectively. Notably, the inflection points for the decline in hepatitis E incidence were 19 years earlier for males than for females in urban areas and four years earlier in rural areas (both P < 0·001). In contrast, no such flection points were observed for bacterial dysentery in either sex. Compared to 2014, the inflection points for the decline in hepatitis E incidence occurred eight years earlier for males and nine years earlier for females (both P < 0·001). Conversely, the inflection points for the decline in acute hemorrhagic conjunctivitis was delayed nine years for males and ten years later for females relative to 2014 (both P < 0·001).

For STBDs, the flection points indicating a decline in incidences of hepatitis C were observed at 67 years of age for males and at 72 years of age in females (both P < 0·001), marking a five-year earlier decline for males compared to females. However, for HIV/AIDS, the flection points were noted at 76 years for males and 65 years for females (both P < 0·001), representing an 11-year later decline for males. Similarly, the flection points for HIV/AIDS occurred two years later for males than for females in urban settings and 11 years later in rural settings (both P < 0·001). Notably, no such flection points were observed for syphilis in either sex across urban and rural areas. Additionally, there was no observed inflection point of decline in syphilis cases among females in 2022, while the inflection points for hepatitis B occurred seven years earlier than in 2014 (both P < 0·001).

For VBZDs, the flection points indicating a decline in the incidences of brucellosis and HFRS were observed at 68 years of age for both sexes (all P < 0·001). Notably, the inflection points indicating a decline in incidences for HFRS occurred two years earlier for males than for females in urban areas and four years earlier in rural areas (both P < 0·001). Furthermore, compared to 2014, the inflection points for the decline in brucellosis incidences in 2022 shifted by nine years later for males and eight years later for females in 2022, while the inflection point for the decline in HFRS incidence advanced by ten years for both sexes (all P < 0·001).

### Changed patterns potentially attributing to COVID-19 pandemic

Compared to the epidemic level during pre-COVID-19 pandemic, a notable reduction in the overall incidence of NIDs among the older people was observed during both Stage I (15·95%) and Stage II (7·62%) of the COVID-19 pandemic. The impact of COVID-19-related NPIs on NIDs varied across different stages of the pandemic. Specifically, for respiratory diseases, the overall incidence decreased by 14·63% in Stage I and 21·74% in Stage II compared to pre-COVID-19 levels. Notably, however, there was an increase in the incidence of seasonal influenza during the initial phase of the pandemic (Stage I). Furthermore, during the initial phase of the COVID-19 pandemic, the overall incidence of GEDs decreased by 35·74%, while that of VBZDs declined by 20·88%. In the subsequent stage of the pandemic (Stage II), GEDs further decreased by 3·49%, while VBZDs increased by 21·09%. Notably, during the Stage II of the COVID-19 pandemic, there was a significant resurgence in the incidence of several diseases, including acute hemorrhagic conjunctivitis (11·76%), infectious diarrhea (4·88%), typhus (30·00%), and brucellosis (37·72%). The incidence of STBDs was suppressed during Stage I of the pandemic but experienced a quick resurgence in Stage II, mainly driven by increases in syphilis (6·76%), HIV/AIDS (4·01%) and hepatitis B (0·83%, Appendix pp 14 and 15).

## Discussion

Between 2014 and 2022, the incidence of respiratory diseases, GEDs and VBZDs in China's older people shows a significant downward trend. This dramatic decline can be attributed to effective large-scale public health interventions and vaccination programs covering a wide range of populations.^6^^,^^20^ However, over the past few years, there has been a significant increase in the incidence of SBTDs‒a phenomenon that has been confirmed in our studies of STBDs in older people. Globally, there has been increasing evidence of the rise in rates of STBDs among the older people.^21^ A variety of factors have been identified as potentially placing older people at risk for STBDs, including sustained but low levels of sexual activity, reduced social stigma and embarrassment, changing norms of sexual behavior, and extramarital relationships resulting primarily from the death or physical condition of a partner.^22^^,^^23^

Geographically, difference was noticeable between regions, with older people in Inner Mongolia-Xinjiang region and South China region continuing to carry a disproportionate burden from NIDs, driven primarily by respiratory diseases and STBDs. Such differences may be related to specific socio-economic, cultural and environmental factors in these regions. Inner Mongolia-Xinjiang region share borders with several countries and have frequent border trade and population movements, increasing the risk of importation and spread of infectious diseases.^24^ In addition, these regions are characterized by a combination of factors that contribute to the growth and spread of infectious diseases‒such as the diverse customs of multi-ethnic populations, limited access to healthcare resources, inefficient public health programs and infrastructure, environmental degradation, and poverty.^6^ South China region is economically developed, densely populated, and rapidly urbanizing, which can lead to more rapid transmission of infectious diseases. Moreover, the warm and humid climate in South China region is suitable for the survival and reproduction of many pathogens, increasing the incidence of infectious diseases.^25^ In addition, there may be differences in the annual incidence reported among different diseases and geographic regions, influenced by the level of detection and intensity of screening.^6^

This study found that the incidence of the categories of infectious diseases was significantly higher in the older people in rural areas than in urban areas.^26^ The higher incidence of infectious diseases among the older people in rural areas may be related to a number of factors, such as an inadequate health insurance system, differences in health-care behaviors, the empty-nest phenomenon and lower socioeconomic status.^27^^,^^28^ Although China has largely achieved universal health insurance coverage, certain regions still exhibit inadequate coverage, which may reduce individuals’ likelihood of promptly seeking medical care.^29^ This delay in healthcare-seeking behavior can exacerbate the transmission of NIDs and enlarge the pool of infected individuals, potentially leading to increased disease incidence, as NIDs are often transmitted by asymptomatic individuals or mild cases who do not access healthcare services.^30^ Therefore, there is a need to strengthen public health services in rural areas, improve sanitary conditions and raise the health awareness of the older people in order to reduce health inequalities between urban and rural areas. Our findings indicated that overall yearly incidence of these four disease categories was significantly higher in males than in females and that there were differences in the incidence of the diseases by age group, with the highest incidence of STBDs and VBZDs in the 60–64 age group, respiratory diseases in the 75–79 age group, and GEDs in the 80–84 age group, suggesting that the burden of infectious diseases is not uniformly distributed in the older people, which provides a quantitative target for precise public health strategies for the older people.

In terms of priority infectious diseases for the older people, from 2014 to 2022, five of the top six NIDs (hepatitis B, syphilis, hepatitis C, infectious diarrhea, and seasonal influenza) in China's older people exhibited an increase in annual incidences, four of which are STBDs. STBDs have become a growing public health problem in the older people. In recent years, the incidence of STBDs has been on the rise globally in the older people aged 60 years and over. This phenomenon is particularly pronounced in certain regions, such as Eastern Europe, Central Asia and the high-income Asia–Pacific region.^31^ Given the global trend of population aging, hepatitis B infection is expected to become more prevalent among the older people. Although effective universal vaccination programs are available, they are predominantly focused on younger populations.^17^ Moreover, the physiological changes associated with aging, and the increasing epidemic level of multimorbidity, have the potential to worsen outcomes in older people patients with chronic hepatitis B infection.^32^ These results suggest that older people patients with chronic hepatitis B infection should be monitored closely and the ever-increasing burden of disease in this population should not be ignored or underestimated. In our study, we observed an age-related increase in the incidence of syphilis in females, a trend that aligns with Brazilian research reporting an elevated detection rate of syphilis among older people.^21^ The number of syphilis cases among 50 years or older have been increasing by nearly eight-fold in Japan in the past 11 years, even among those aged ≥70 years.^33^ A previous study showed a positive correlation between the incidence of syphilis and the proportion of older people.^34^ The global incidence of hepatitis C virus infection is estimated to be about 0·7% of the world's population, corresponding to 56·8 million infections.^35^ Hepatitis C infection disproportionately affects different generations, with an important peak among people born between 1945 and 1965 (the “baby boomer” generation), this is consistent with our findings.^36^

Due to age-related physiologic changes in the gastrointestinal tract, older people are particularly susceptible to infectious diarrhea e.g., norovirus infection. Enteric pathogens are typically acquired through fecal-oral transmission from potential reservoirs such as hospitals, nursing homes, and long-term care facilities.^37^ Decreased gastric acid secretion associated with aging compromises the stomach's barrier function against ingested pathogens. Additionally, immunosenescence leads to reduced secretion of immunoglobulin A, further elevating the risk of gut-specific infections.^38^ Seasonal influenza, which predominates in the older age group of 80–84 years, declined in incidence during COVID-19 pandemic but then rebounded sharply. Currently, vaccination continues to be the cornerstone of influenza prevention. Several national and international studies have shown that during the flu season, influenza vaccination in older people prevents 28% of flu-related complications, 39% of flu-like symptoms, and 49% of confirmed influenza.^39^ Despite the efficacy of influenza vaccines, existing vaccination programs provide suboptimal protection, likely due to a widespread prevalence of vaccine hesitancy among residents, especially among well-educated and urban-dwelling older people individuals. No such flection points of decreasing seasonal influenza incidence with age were observed in either male or female patients, with the age-specific incidence of seasonal influenza exhibiting a linear increase with advancing age. This means that both males and females have an increased risk of getting the seasonal influenza as they age. All sectors of society should pay more attention to the older people and provide the necessary health education and protection support. Although tuberculosis incidence declined significantly by an average of 6·4% per year (APC, P < 0·001), it remains highest among older people. This downward trend is closely linked to the gradual expansion of national tuberculosis control program since the 1990s, which achieved full coverage in 2000.^40^ However, with accelerated population aging, the resurgence of latent tuberculosis infection is becoming a new challenge: immune senescence is a central driver of resurgence, and viral or bacterial infections further amplify immune dysfunction and inflammatory responses.^41^^,^^42^ Therefore, for tuberculosis control among the older people, the current priority should be on case finding, treatment, and TPT (as appropriate). In addition, vaccination should be considered a key complementary strategy once available.^43^

The advent of the COVID-19 pandemic had caused unexpected shifts in the epidemic trends of infectious diseases among the older people. Several studies have demonstrated a decrease in the incidences of most infectious diseases during the COVID-19 pandemic, due to the implementation of strict NPIs.^14^^,^^44^^,^^45^ GEDs and VBZDs were the diseases that showed the most marked decline in the older people during the COVID-19 pandemic Stage I. The NPIs implemented in response to COVID-19 have significantly restricted the frequency and scope of activities among the older people, thereby reducing the risk of the older people encountering infectious sources.^46^^,^^47^ However, due to weakened immune function and various underlying diseases, the older people may experience an unusually rapid rebound of infectious diseases following the relaxation of NPIs. Notably, the incidence of seasonal influenza increased significantly in the older people during COVID-19 pandemic Stage I. However, other studies in non-older people showed that the respiratory diseases experiencing the most substantial declines, especially seasonal influenza.^48^, ^49^, ^50^ Possible reasons for this were that the older people was at high risk for COVID-19, making them more susceptible to the double attack of seasonal influenza and COVID-19 due to their compromised immunity.^51^ Alternatively, the high intensity of screening might have raised the reported rate of older people contracting seasonal influenza. Our study provides new evidence indicating that the implementation of national restrictions in response to the COVID-19 pandemic did not consistently or uniformly reduce the incidence of respiratory diseases among the older people.

The limitations of our study should be acknowledged. First, the annual reported incidence, based on the data from the reporting system, may be subject to underestimation due to varying screening intensities. This variation in screening intensity across different regions can lead to underreporting of cases, particularly in areas with limited resources and diagnostic capabilities. Second, selection bias may also lead to underestimation of incidence, as individuals with specific infectious diseases may be more likely to avoid screening compared to those without infections. This bias can result in a lower reported incidence than the true incidence in the population. Third, the incidence of NIDs during the COVID-19 pandemic is influenced by a multitude of factors. Another important limitation of this study is that it does not include data from the post-COVID-19 period (2023–2024). This restricts our comprehensive understanding of the epidemiological characteristics of infectious diseases post-COVID-19 pandemic. In future research, we will concentrate on analyzing the epidemiological features of NIDs among older people in the post-COVID-19 era. This focus will provide valuable scientific support for the management of infectious diseases in this vulnerable population.

In conclusion, apart from STBDs, the incidences of all the other categories exhibited a decreasing trend. STBDs such as hepatitis B, syphilis, hepatitis C, and HIV/AIDS have increased in incidence prior to COVID-19 and require continued attention. Expand vaccination of seniors against hepatitis B, and seasonal influenza, prevent further escalation of these diseases; and redouble efforts to combat major diseases in seniors, including hepatitis B, infectious diarrhea, tuberculosis, syphilis, seasonal influenza, and hepatitis C. Personalized and precise strategies for prevention and control of these diseases should be applied for aged 60–70 years, males, and people living in specific regions.

## Contributors

Conceptualization: SHL, CLL, LPW, PTB, LQF, WL; Methodology: SHL, CLL, MJG, RCG, LPW, PTB, LQF, WL; Data collection: SHL, MJG, YQS, YHW, YMZ, TT, CXS, YT, YBQ, JM, YZ and YFY; Visualization: SHL, CLL, QX, GLW; Supervision: QL, YPZ, LQF, WL, LPW, PTB; Draft writing: SHL; Manuscript review & editing: LQF, WL, CLL, LPW, PTB.

## Data sharing statement

Raw data of reported cases for each notifiable infectious diseases are not publicly available and are protected due to data privacy laws. Deidentified and aggregated data may be requested from the corresponding author (Dr. Li-Qun Fang) with permission from the data provider (Dr. Li-Ping Wang).

## Editor note

The Lancet Group takes a neutral position with respect to territorial claims in published maps and institutional affiliations.

## Declaration of interests

Authors declare that they have no competing interests.

## Appendix A Supplementary data

Supplementary Appendix

Translated Abstract-Fang

## References

[1] Beard J.R., Officer A., de Carvalho I.A. (2016). The world report on ageing and health: a policy framework for healthy ageing. Lancet.

[2] Fang E.F., Xie C., Schenkel J.A. (2020). A research agenda for ageing in China in the 21st century (2nd edition): focusing on basic and translational research, long-term care, policy and social networks. Ageing Res Rev.

[3] United Nations (2022). Department of Economic and Social Affairs, Population Division. World population prospects 2022: data sources. https://digitallibrary.un.org/record/3992030?ln=zh_CN&v=pdf.

[4] World Health Organization (2018). World health statistics 2018: monitoring health for the SDGs, sustainable development goals report. http://apps.who.int/iris/bitstream/handle/10665/272596/9789241565585-eng.pdf?ua=1.

[5] World Health Organization (2015). China country assessment report on ageing and health. https://iris.who.int/handle/10665/194271.

[6] Yang S., Wu J., Ding C. (2017). Epidemiological features of and changes in incidence of infectious diseases in China in the first decade after the SARS outbreak: an observational trend study. Lancet Infect Dis.

[7] Chinese Center for Disease Control and Prevention (2025). Infectious diseases. https://www.chinacdc.cn/jkyj/crb2/.

[8] Møgelmose S., Neels K., Beutels P., Hens N. (2023). Exploring the impact of population ageing on the spread of emerging respiratory infections and the associated burden of mortality. BMC Infect Dis.

[9] Li S.J., Li Y.F., Song W.M. (2021). Population aging and trends of pulmonary tuberculosis incidence in the elderly. BMC Infect Dis.

[10] Zhao N., Wang S., Wang L. (2023). Epidemiological features and trends in the mortality rates of 10 notifiable respiratory infectious diseases in China from 2004 to 2020: based on national surveillance. Front Public Health.

[11] Dong K., Gong H., Zhong G. (2023). Estimating mortality associated with seasonal influenza among adults aged 65 years and above in China from 2011 to 2016: a systematic review and model analysis. Influenza Other Respir Viruses.

[12] United Nations, Department of Economic and Social Affairs, Population Division (2019). World population ageing 2019-highlights. https://digitallibrary.un.org/record/3846855.

[13] China National People's Congress website (2012). Law of the People's Republic of China on the protection of the rights and interests of the elderly. http://www.npc.gov.cn/zgrdw/huiyi/cwh/1130/2012-12/29/content_1749760_2.htm.

[14] Geng M.-J., Zhang H.-Y., Yu L.-J. (2021). Changes in notifiable infectious disease incidence in China during the COVID-19 pandemic. Nat Commun.

[15] Dong Y., Wang L., Burgner D.P. (2020). Infectious diseases in children and adolescents in China: analysis of national surveillance data from 2008 to 2017. BMJ.

[16] World Health Organization (2025). Guidelines for the prevention, care and treatment of persons with chronic hepatitis B infection. https://www.who.int/publications/i/item/9789241549059.

[17] Kang C.K., Brennan P.N., Dillon J.F. (2022). How to effectively monitor aging patients with chronic hepatitis B: a review. Clin Interv Aging.

[18] Kim H.J., Fay M.P., Feuer E.J., Midthune D.N. (2000). Permutation tests for joinpoint regression with applications to cancer rates. Stat Med.

[19] (2022). Statistical methodology and applications branch SRP, National Cancer Institute.

[20] Wang L., Wang Y., Jin S. (2008). Emergence and control of infectious diseases in China. Lancet.

[21] Barros Z.D.S., Rodrigues B.G.M., Frota K.M.G. (2023). Syphilis detection rate trend in aged people: Brazil, 2011-2019. Rev Bras Epidemiol.

[22] Kumar B., Kaushal I., Narayanan B., Narang T. (2025). Sexually transmitted infections in the elderly: a growing concern in geriatric care. Indian J Sex Transm Dis AIDS.

[23] Ševčíková A., Sedláková T. (2020). The role of sexual activity from the perspective of older adults: a qualitative study. Arch Sex Behav.

[24] Zhang Q., Sun J., Zhang Z. (2016). Risk assessment of malaria in land border regions of China in the context of malaria elimination. Malar J.

[25] Yang M., Chen C., Zhang X. (2022). Meteorological factors affecting infectious diarrhea in different climate zones of China. Int J Environ Res Public Health.

[26] Chen L., Xing Y., Zhang Y. (2024). Long-term variations of urban-rural disparities in infectious disease burden of over 8.44 million children, adolescents, and youth in China from 2013 to 2021: an observational study. PLoS Med.

[27] Wang G., Hu M., Xiao S.Y., Zhou L. (2017). Loneliness and depression among rural empty-nest elderly adults in Liuyang, China: a cross-sectional study. BMJ Open.

[28] Shahar S., Vanoh D., Mat Ludin A.F., Singh D.K.A., Hamid T.A. (2019). Factors associated with poor socioeconomic status among Malaysian older adults: an analysis according to urban and rural settings. BMC Public Health.

[29] Smith K.T., Monti D., Mir N., Peters E., Tipirneni R., Politi M.C. (2018). Access is necessary but not sufficient: factors influencing delay and avoidance of health care services. MDM Policy Pract.

[30] Yu L.J., Ji P.S., Ren X. (2025). Inter-city movement pattern of notifiable infectious diseases in China: a social network analysis. Lancet Reg Health West Pac.

[31] Fu L., Tian T., Wang B. (2024). Global, regional, and national burden of HIV and other sexually transmitted infections in older adults aged 60-89 years from 1990 to 2019: results from the global burden of disease study 2019. Lancet Healthy Longev.

[32] Mohammed Abdul M.K., Snyder H.S., Chunduru M., Lee S.M.K., Satapathy S.K. (2020). Hepatitis C virus in the elderly in the direct-acting antiviral era: from diagnosis to cure. Curr Treat Options Infect Dis.

[33] Takahashi M., Hagiya H., Koyama T., Otsuka F. (2022). Trends in the incidence of syphilis in the middle-aged and older adults in Japan: a nationwide observational study, 2009–2019. Geriatr Gerontol Int.

[34] Zhu X., Zhu Z., Gu L. (2022). Spatio–temporal variation on syphilis from 2005 to 2018 in Zhejiang Province, China. Front Public Health.

[35] Polaris Observatory HCV Collaborators (2022). Global change in hepatitis C virus prevalence and cascade of care between 2015 and 2020: a modelling study. Lancet Gastroenterol Hepatol.

[36] Qureshi K., Petersen T., Andres J. (2020). Hepatitis C treatment differences in elderly patients: single-center retrospective study. Ann Pharmacother.

[37] Dawod E., Crawford C.V. (2021). Common diarrheal illnesses in the elderly. Clin Geriatr Med.

[38] Sato S., Kiyono H., Fujihashi K. (2015). Mucosal immunosenescence in the gastrointestinal tract: a mini-review. Gerontology.

[39] Beyer W.E., McElhaney J., Smith D.J., Monto A.S., Nguyen-Van-Tam J.S., Osterhaus A.D. (2013). Cochrane re-arranged: support for policies to vaccinate elderly people against influenza. Vaccine.

[40] Wang L., Zhang H., Ruan Y. (2014). Tuberculosis prevalence in China, 1990–2010; a longitudinal analysis of national survey data. Lancet.

[41] Allué-Guardia A., Torrelles J.B., Sigal A. (2023). Tuberculosis and COVID-19 in the elderly: factors driving a higher burden of disease. Front Immunol.

[42] Lee K.-A., Flores R.R., Jang I.H., Saathoff A., Robbins P.D. (2022). Immune senescence, immunosenescence and aging. Front Aging.

[43] World Health Organization (2023). Global tuberculosis report 2023. https://www.who.int/publications/i/item/9789240083851.

[44] Li Z.J., Yu L.J., Zhang H.Y. (2022). Broad impacts of coronavirus disease 2019 (COVID-19) pandemic on acute respiratory infections in China: an observational study. Clin Infect Dis.

[45] Adegbija O., Walker J., Smoll N., Khan A., Graham J., Khandaker G. (2018). Notifiable diseases after implementation of COVID-19 public health prevention measures in Central Queensland, Australia. Commun Dis Intell.

[46] Braunstein S.L., Slutsker J.S., Lazar R. (2021). Epidemiology of reported HIV and other sexually transmitted infections during the COVID-19 pandemic, New York City. J Infect Dis.

[47] Ullrich A., Schranz M., Rexroth U. (2021). Impact of the COVID-19 pandemic and associated non-pharmaceutical interventions on other notifiable infectious diseases in Germany: an analysis of national surveillance data during week 1-2016 - week 32-2020. Lancet Reg Health Eur.

[48] Chen L., Wang L., Xing Y. (2024). Persistence and variation of the indirect effects of COVID-19 restrictions on the spectrum of notifiable infectious diseases in China: analysis of national surveillance among children and adolescents from 2018 to 2021. JMIR Public Health Surveill.

[49] Chen J.M., Chen Y.Q., Sun Y.X. (2022). Control of COVID-19 in China likely reduced the burden of multiple other infectious diseases. J Infect.

[50] Kadambari S., Goldacre R., Morris E., Goldacre M.J., Pollard A.J. (2022). Indirect effects of the covid-19 pandemic on childhood infection in England: population based observational study. BMJ.

[51] Liang J., Wang Y., Lin Z. (2024). Influenza and COVID-19 co-infection and vaccine effectiveness against severe cases: a mathematical modeling study. Front Cell Infect Microbiol.

