# Supplementary material for: Epidemiological analysis of infectious diseases in older people in China from 2014 to 2022: a population-based study
Source: Lancet Reg Health West Pac. 2025 Nov 3;64:101729. doi: 10.1016/j.lanwpc.2025.101729 (PMC12677096; doi:10.1016/j.lanwpc.2025.101729)

Supplementary appendix

Supplement to: Epidemiological analysis of infectious diseases in older people in China from 2014 to 2022: a population-based study

Table of Contents

Page	Item
2	Supplementary Text
2	Data collection and management.
3	Nonpharmaceutical interventions (NPIs) implemented during the COVID-19 pandemic
4	Supplemental Reference
5	Supplemental Tables
5	Table S1. The 21 major NIDs included in this study.
6—7	Table S2. Differences in the incidence of NIDs between the older people and younger adults.
8	Table S3. Proportion of NIDs cases among the older people in China, 2014–2022.
9	Table S4. Incidence (1/100 000) of acute and chronic infectious diseases stratified by categories among the older people in China, 2014–2022.
10	Table S5. Case number and proportion of various types of syphilis among the older people in China, 2014–2022.
11—13	Table S6. The estimated inflection points (95% CI) of age at NIDs among the older people in China, 2014–2022.
14—15	Table S7. Comparison of crude average monthly incidences (1/100 000) of 21 NIDs between pre-COVID-19 pandemic and COVID-19 pandemic in China.
16	Supplemental Figures
16	Figure S1. Flow chart of the data processing procedure.
17	Figure S2. Ranking of 43 infectious diseases among different populations in China, 2014–2022.
18	Figure S3. Geographical distribution of seven ecological zones in the mainland of China.
19	Figure S4. Overall temporal trends in incidences of four categories of infectious diseases among the older people in China, 2014–2022.
20	Figure S5. Overall temporal trends in incidences of 21 NIDs among the entire population in China, 2014–2022.
21	Figure S6. Seasonal distribution of the incidences for 21 NIDs among the older people in China, 2014–2022.
22	Figure S7. Ranking of incidence of each of the 21 NIDs by age group, from 2014 to 2022.
23	Figure S8. Infectious diseases with greatest incidence by age, 2014–2022.
24	Figure S9. Changing trends in the age-specific incidences of other six NIDs among the older people, stratified by sex, as analyzed using Join-Point regression model.
25—26	Figure S10. Changing trends in the age-specific incidences of infectious diseases among the older people, stratified by urban areas, as analyzed using Join-Point regression model.
27—28	Figure S11. Changing trends in the age-specific incidences of infectious diseases among the older people, stratified by rural areas, as analyzed using Join-Point regression model.
29—30	Figure S12. Changing trends in the age-specific incidences of infectious diseases among the older people, stratified by year, as analyzed using Join-Point regression model.

Supplementary Text

Data collection and management

The surveillance system and classification of the notifiable infectious diseases (NIDs) in the China Information System for Disease Control and Prevention (CISDCP), all NIDs were diagnosed according to their standard diagnostic criteria.[1,2] Only patients with confirmed diagnosis (lab-confirmed and clinically confirmed cases) were included in the analysis and the suspected cases were excluded. According to the protocol of reporting NIDs, each diagnosed case is assigned a unique resident ID number in the CISDCP. Demographic data such as sex, age, and occupation, clinical data such as dates of symptom onset, diagnosis, hospital visit, and death, and laboratory results (if available) are first logged into hospital electronic health record systems by physicians or trained hospital staff, which are reviewed and formally uploaded to the CISDCP by the staff. By 2022, there are currently 40 NIDs in China, which are divided into the following three classes: Category A (two diseases) infectious diseases are those that pose an extremely severe threat to human health and life, potentially causing significant economic losses and social impact. These diseases require the strictest control measures to prevent the spread of outbreaks. They include: plague and cholera; Category B (27 diseases) infectious diseases are those that pose a severe threat to human health and life, potentially causing considerable economic losses and social impact. These diseases require stringent management to reduce incidence rates and mitigate harm. They include: SARS, acquired immune deficiency syndrome (AIDS), hepatitis (A, B, D, C, E, and untyped), poliomyelitis, human infection with highly pathogenic avian influenza, , measles, hemorrhagic fever with renal syndrome, rabies, japanese encephalitis, dengue, anthrax, dysentery, tuberculosis, typhoid and paratyphoid, epidemic cerebrospinal meningitis, pertussis, diphtheria, neonatal tetanus, scarlet fever, brucellosis, gonorrhoea, syphilis, leptospirosis, schistosomiasis, malaria, H7N9, COVID-19 (added in 2020); Category C (11 diseases) infectious diseases are those that are common and frequently occurring, posing a threat to human health and life, potentially causing some degree of economic losses and social impact. These diseases require monitoring of epidemic trends and control of outbreaks. They include: seasonal influenza, mumps, rubella, acute haemorrhagic conjunctivitis, leprosy, typhus, kala-azar, hydatid disease, filariasis, HFMD (hand, foot, and mouth disease), infectious diarrhea (infectious diarrhea excludes cholera, dysentery, typhoid and paratyphoid).[3,4]

In this study, viral hepatitis is further grouped into hepatitis A, hepatitis B, hepatitis C, hepatitis D, hepatitis E and hepatitis (untyped), transmission routes and clinical manifestations of which vary. Similarly, dysentery is divided into bacterial dysentery and amoebic dysentery, respectively. All laboratory-confirmed cases and clinically diagnosed cases were included in the analysis between January 1, 2014 and December 31, 2022. In total, 46 kinds of NIDs were reported in CISDCP. We first quantified the relative burden of disease in older people, then excluded cases under 60 years of age and included diseases for which older people reported more than 100 cases per year during the study period (23 diseases, including dengue, schistosomiasis, japanese encephalitis, leptospirosis, leprosy, hepatitis D, H7N9, rubella, malaria, measles, anthrax, pertussis, kala-azar, scarlet fever, cholera, plague, epidemic cerebrospinal meningitis, human infection with highly pathogenic avian influenza, poliomyelitis, diphtheria, filariasis, neonatal tetanus, and SARS were excluded because of less than 100 cases per year during the study period). Hepatitis (untyped) and COVID-19 were excluded. Finally, 21 NIDs were remained and grouped into four categories based on the mode of transmission of each infectious disease (Appendix p 5): respiratory diseases; gastrointestinal or enteroviral diseases (GEDs);

sexually transmitted or bloodborne diseases (STBDs); and vector-borne or zoonotic diseases (VBZDs).[1,2]

Nonpharmaceutical interventions (NPIs) implemented during the COVID-19 pandemic

We divided the nine-year study period into three stages, pre-COVID-19 pandemic (Jan 1, 2014–Jan 22, 2020), COVID-19 pandemic Stage I (Jan 23, 2020–Apr 30, 2020), COVID-19 pandemic Stage II (May 1, 2020–Nov 30, 2022), based on the temporal characteristics of the COVID-19 pandemic.[5] In COVID-19 pandemic Stage I, the most intense COVID-19 restrictions were implemented, including stay-at-home or shelter-in-place order, closure of nonessential businesses, restaurants, schools and hotels, prohibition of gatherings, etc. In COVID-19 pandemic Stage II, on April 29, 2020, China entered the stage of normalized prevention and control of the COVID-19 pandemic, and on May 7, 2020, the State Council's Joint Prevention and Control Mechanism issued the Guiding Opinions on Doing a Good Job of Normalized Prevention and Control of the COVID-19 pandemic. During this phase, imported cases from abroad were basically brought under control, and the overall local epidemic in China was sporadic, with occasional small-scale localized aggregation of epidemics, all of which were rapidly and effectively brought under control, with the positive trend of the epidemic continuing to be consolidated and socio-economic life rapidly returning to normal.[6] On December 7, 2022, the Joint Prevention and Control Mechanism of the State Council issued the "Notice on Further Optimizing and Implementing COVID-19 Prevention and Control Measures", which gradually phased out non-pharmaceutical interventions. Therefore, we excluded the data from December 2022.[7]

For each disease, the percentage of change (PC) in incidence was calculated as follows:

$$\frac{i_{time_1}(p) - i_{time_2}(p)}{i_{time_2}(p)} \times 100\%$$

where $time_1$ refers to either Stage I or Stage II of the COVID-19 pandemic, while $time_2$ indicates the pre-COVID-19 pandemic. $i_{time_1}(p)$ represent the incidence or average incidence during phase p within $time_1$, and $i_{time_2}(p)$ represents the incidence or average incidence specific to phase p within $time_2$.

Supplemental Reference

1 Geng MJ, Zhang HY, Yu LJ, et al. Changes in notifiable infectious disease incidence in China during the COVID-19 pandemic. *Nat Commun* 2021; **12:** 6923.

2 Dong Y, Wang L, Burgner DP, et al. Infectious diseases in children and adolescents in China: analysis of national surveillance data from 2008 to 2017. *BMJ* 2020; **369:** m1043.

3 Chinese Center for Disease Control and Prevention. Infectious diseases. 2016. https://www.chinacdc.cn/jkyj/crb2/202409/t20240906_297034.html. Accessed July 16, 2025.

4 The Supreme People's Procuratorate of the People's Republic of China. Law of the People's Republic of China on prevention and control of infectious diseases. 2025. https://www.spp.gov.cn/spp/zdgz/202504/t20250430_694756.shtml. Accessed July 16, 2025.

5 Chen L, Wang L, Xing Y, et al. Persistence and variation of the indirect effects of COVID-19 restrictions on the spectrum of notifiable infectious diseases in China: analysis of national surveillance among children and adolescents from 2018 to 2021. *JMIR Public Health Surveill* 2024; **10:** e47626.

6 State Council Responds to Joint Prevention and Control Mechanism for Novel Coronavirus-Infected Pneumonia Epidemic. China: Guiding opinions on doing a good job of normalized prevention and control of the COVID-19 pandemic. 2020. https://www.gov.cn/zhengce/content/2020-05/08/content_5509896.htm. Accessed July 16, 2025.

7 State Council Responds to Joint Prevention and Control Mechanism for Novel Coronavirus-Infected Pneumonia Epidemic. Notice on further optimizing the implementation of preventive and control measures for the new crown pneumonia epidemic. 2022. https://www.nhc.gov.cn/xcs/gzzcwj/202212/15457b5897ba42c991e3ee3b1f1f4bed.shtml. Accessed July 16, 2025.

Supplemental Tables

Table S1. The 21 major NIDs included in this study.

Disease		Abbreviation
**Respiratory diseases**		
1	Tuberculosis	TB*
2	Mumps	Mumps
3	Seasonal influenza	Flu
**Gastrointestinal or enteroviral diseases**		GEDs
4	Hepatitis A	Hepatitis A
5	Hepatitis E	Hepatitis E
6	Hand, foot and mouth disease	HFMD
7	Typhoid and paratyphoid	T/P
8	Bacterial dysentery	BD
9	Acute hemorrhagic conjunctivitis	AHC
10	Infectious diarrhea other than cholera, dysentery, typhoid and paratyphoid	ID
11	Amoebic dysentery	AD
**Sexually transmitted or bloodborne diseases**		STBDs
12	Gonorrhea	Gonorrhea
13	Syphilis	Syphilis*
14	HIV/AIDS	HIV/AIDS*
15	Hepatitis B	Hepatitis B*
16	Hepatitis C	Hepatitis C*
**Vector-borne or zoonotic diseases**		VBZDs
17	Typhus	Typhus
18	Brucellosis	Brucellosis*
19	Hemorrhagic fever with renal syndrome	HFRS
20	Rabies	Rabies
21	Hydatid disease	Hydatid disease*

*These seven infectious diseases are categorized as chronic infectious diseases, while the remaining 14 infectious diseases are categorized as acute infectious diseases.

Table S2. Differences in the incidence of NIDs between the older people and younger adults.

Disease	Older people (aged≥60 years)		Younger adults (18–59 years old)		IRR (Older people vs. Younger adults)		
	Cases	Incidence (1/100 000)	Cases	Incidence (1/100 000)		95%CI	*p*-value
Tuberculosis	2 421 363	101·902	4 419 899	57·906	1·760	1·757, 1·763	<0·001
Hepatitis B	1 800 188	75·760	6 677 355	87·481	0·866	0·865, 0·867	<0·001
Infectious diarrhea	1 225 650	51·581	2 735 362	35·836	1·439	1·436, 1·442	<0·001
Syphilis	1 206 976	50·795	2 816 215	36·896	1·377	1·374, 1·380	<0·001
Hepatitis C	563 107	23·698	1 215 591	15·926	1·488	1·483, 1·493	<0·001
Seasonal influenza	560 259	23·578	2 175 636	28·503	0·827	0·825, 0·830	<0·001
HIV/AIDS	274 522	11·553	1 004 809	13·164	0·878	0·874, 0·881	<0·001
Bacterial dysentery	157 396	6·624	329 672	4·319	1·534	1·524, 1·543	<0·001
Brucellosis	100 180	4·216	357 131	4·679	0·901	0·895, 0·907	<0·001
Hepatitis E	76 794	3·232	142 410	1·866	1·732	1·717, 1·747	<0·001
AHC	60 194	2·533	168 934	2·213	1·145	1·134, 1·155	<0·001
Gonorrhea	47 246	1·988	943 794	12·365	0·161	0·159, 0·162	<0·001
Hepatitis A	39 172	1·649	90 767	1·189	1·387	1·370, 1·403	<0·001
Hepatitis (untyped)	30 755	1·294	96 720	1·267	1·021	1·008, 1·035	0·001
HFRS	21 501	0·905	61 563	0·807	1·121	1·105, 1·139	<0·001
Mumps	19 471	0·819	178 153	2·334	0·351	0·346, 0·356	<0·001
T/P	15 698	0·661	42 359	0·555	1·191	1·169, 1·212	<0·001
Dengue	14 630	0·616	67 877	0·889	0·693	0·680, 0·705	<0·001
Schistosomiasis	12 611	0·531	34 599	0·453	1·172	1·147, 1·195	<0·001
Hydatid disease	7 177	0·302	25 773	0·338	0·893	0·871, 0·918	<0·001
Typhus	2 774	0·117	5 983	0·078	1·500	1·424, 1·558	<0·001
Rabies	1 672	0·070	1 916	0·025	2·803	2·625, 2·993	<0·001
HFMD	1 430	0·060	87 543	1·147	0·052	0·050, 0·055	<0·001
Japanese encephalitis	1 514	0·064	2 198	0·029	2·207	2·072, 2·362	<0·001
Amoebic dysentery	1 294	0·054	2 671	0·035	1·543	1·456, 1·663	<0·001
Leptospirosis	985	0·041	1 755	0·023	1·783	1·668, 1·949	<0·001
Leprosy	671	0·028	2 516	0·033	0·848	0·787, 0·933	<0·001
Hepatitis D	534	0·022	1 643	0·022	1·000	0·947, 1·151	0·387
H7N9	479	0·020	642	0·008	2·500	2·129, 2·698	<0·001
Rubella	408	0·017	25 832	0·338	0·050	0·046, 0·056	<0·001
Malaria	396	0·017	19 862	0·260	0·065	0·058, 0·071	<0·001
Measles	389	0·016	53 955	0·707	0·023	0·021, 0·026	<0·001
Anthrax	340	0·014	2 470	0·032	0·438	0·395, 0·495	<0·001
Pertussis	274	0·012	1 579	0·021	0·571	0·490, 0·634	<0·001
Kala-azar	271	0·011	781	0·010	1·100	0·971, 1·280	0·124
Scarlet fever	157	0·007	5 301	0·069	0·101	0·081, 0·112	<0·001
Cholera	38	0·002	144	0·002	1·000	0·593, 1·212	0·365
ECM	27	0·001	176	0·002	0·500	0·329, 0·739	0·001
Plague	3	0·000*	14	0·000*	0·688	0·198, 2·395	0·557

HIHPAI	1	0·000*	5	0·000*	0·642	0·075, 5·499	0·686
Diphtheria	1	0·000*	2	0·000*	1·606	0·146, 17·714	0·699
Filariasis	-	-	1	0·000*	0·000	-	<0·001
Total	8 668 548	364·812	23 801 608	311·827	1·170	1·169, 1·171	<0·001

Note: Exclude COVID-19, poliomyelitis, SARS, and neonatal tetanus. HFRS: hemorrhagic fever with renal syndrome; T/P: typhoid and paratyphoid; HFMD: hand, foot and mouth disease; AHC: acute hemorrhagic conjunctivitis; ECM: epidemic cerebrospinal meningitis; HIHPAI: human infection with highly pathogenic avian influenza.*indicates that the value is too small, ranging between 0.00001 and 0.0002.

Table S3. Proportion of NIDs cases among the older people in China, 2014–2022.

Diseases	Cases	Incidence (1/100 000)	Percent (%)
**Respiratory diseases**	**3 001 093**	**126·30**	**34·88**
Tuberculosis	2 421 363	101·90	28·14
Mumps	19 471	0·82	0·23
Seasonal influenza	560 259	23·58	6·51
**Gastrointestinal or enteroviral diseases**	**1 577 628**	**66·39**	**18·35**
Hepatitis A	39 172	1·65	0·46
Hepatitis E	76 794	3·23	0·89
Hand, foot and mouth disease	1 430	0·06	0·02
Typhoid and paratyphoid	15 698	0·66	0·18
Bacterial dysentery	157 396	6·62	1·83
Acute hemorrhagic conjunctivitis	60 194	2·53	0·70
Infectious diarrhea	1 225 650	51·58	14·25
Amoebic dysentery	1 294	0·06	0·02
**Sexually transmitted or bloodborne diseases**	**3892 039**	**163·80**	**45·23**
Gonorrhea	47 246	1·99	0·55
Syphilis	1 206 976	50·80	14·03
HIV/AIDS	274 522	11·55	3·19
Hepatitis B	1 800 188	75·76	20·92
Hepatitis C	563 107	23·70	6·54
**Vector-borne or zoonotic diseases**	**133 304**	**5·61**	**1·54**
Typhus	2 774	0·12	0·03
Brucellosis	100 180	4·22	1·16
Hemorrhagic fever with renal syndrome	21 501	0·90	0·25
Rabies	1 672	0·07	0·02
Hydatid disease	7 177	0·30	0·08
**Leading diseases***	**7 777 543**	**327·32**	**90·39**
**Total**	**8 604 064**	**362·10**	**100·00**

*Includes tuberculosis, hepatitis B, infectious diarrhea, syphilis, hepatitis C, and seasonal influenza.

Table S4. Incidence (1/100 000) of acute and chronic infectious diseases stratified by categories among the older people in China, 2014–2022.

Provinces	Respiratory diseases		GEDs	STBDs		VBZDs	
	**Acute**	**Chronic**	**Acute**	**Acute**	**Chronic**	**Acute**	**Chronic**
Beijing	107·28	47·15	110·42	0·69	40·27	0·06	0·94
Tianjin	15·36	40·00	189·02	0·44	40·44	0·18	1·44
Hebei	42·53	62·50	80·70	0·48	109·73	0·93	7·92
Shanxi	21·73	62·71	67·79	1·03	196·59	0·12	20·63
Inner Mongolia	9·55	94·08	13·73	1·24	165·05	0·54	49·58
Liaoning	3·77	60·90	39·27	0·97	96·99	1·83	6·14
Jilin	3·33	60·70	5·47	0·82	64·92	1·63	3·69
Heilongjiang	3·79	96·47	11·45	0·64	67·50	3·13	9·72
Shanghai	18·56	34·22	19·30	5·35	109·25	0·02	0·04
Zhejiang	69·11	73·00	109·18	7·68	80·71	0·74	0·30
Jiangsu	8·24	56·37	17·88	2·90	60·22	0·51	0·28
Anhui	33·59	117·52	186·10	2·09	207·57	0·98	0·33
Shandong	12·47	47·21	98·70	0·77	105·65	1·55	4·30
Henan	29·29	98·16	76·31	0·83	152·21	0·52	6·47
Hubei	28·46	113·50	69·93	1·42	216·19	1·64	0·40
Hunan	33·78	171·64	31·95	1·78	259·22	1·62	0·26
Jiangxi	28·20	152·91	84·04	3·14	181·33	1·97	0·18
Fujian	17·30	83·98	42·94	3·84	258·21	1·51	0·41
Guangdong	39·27	141·65	65·03	3·36	314·30	0·56	0·54
Guangxi	15·31	184·82	146·18	2·42	226·19	0·47	0·33
Hainan	19·79	175·26	50·61	3·69	303·37	0·29	0·22
Chongqing	21·41	112·03	63·87	2·94	202·03	0·14	0·13
Sichuan	13·27	78·67	30·54	1·38	174·01	0·32	0·73
Yunnan	15·24	100·83	49·39	1·11	149·13	1·63	1·13
Guizhou	9·31	191·78	27·81	1·77	206·40	0·28	0·19
Tibet	1·97	230·81	19·11	0·39	124·09	0·04	4·86
Shaanxi	20·82	76·84	32·88	0·80	142·44	5·92	3·86
Ningxia	13·25	117·64	123·88	1·52	210·00	0·16	60·91
Gansu	26·33	113·47	68·96	0·91	171·91	0·43	14·74
Qinghai	13·31	233·30	46·40	1·20	346·52	0·03	27·00
Xinjiang	10·08	688·28	58·04	1·37	428·45	0·12	32·68
**Total**	**24·40**	**101·90**	**66·39**	**1·99**	**161·81**	**1·09**	**4·52**

Table S5. Case number and proportion of various types of syphilis among the older people in China, 2014–2022.

Year	Syphilis	Latent Syphilis		Primary Syphilis		Secondary Syphilis		Tertiary Syphilis	
	Cases	Cases	Percent (%)	Cases	Percent (%)	Cases	Percent (%)	Cases	Percent (%)
2014	77 415	53 209	68·73	16 145	20·86	7 269	9·39	792	1·02
2015	86 828	64 515	74·30	14 243	16·40	7 316	8·43	754	0·87
2016	121 691	100 947	82·95	12 561	10·32	6 985	5·74	1 198	0·99
2017	140 244	119 475	85·19	12 763	9·10	6 899	4·92	1 107	0·79
2018	152 702	132 500	86·77	11 952	7·83	6 982	4·57	1 268	0·83
2019	184 562	165 847	89·86	11 021	5·97	6 380	3·46	1 314	0·71
2020	159 709	146 216	91·55	7 825	4·90	4 598	2·88	1 070	0·67
2021	147 860	136 063	92·02	6 720	4·54	4 128	2·80	949	0·64
2022	135 965	127 099	93·48	4 999	3·68	3 058	2·25	809	0·59
**Total**	**1 206 976**	**1 045 871**	**86·65**	**98 229**	**8·14**	**53 615**	**4·44**	**9 261**	**0·77**

Table S6. The estimated inflection points (95% CI) of age at NIDs among the older people in China, 2014–2022.

Diseases	Estimated inflection points (95% CI)								
	Total			Urban areas			Rural areas		
	Male	Female	Estimated point difference	Male	Female	Estimated point difference	Male	Female	Estimated point difference
**Respiratory diseases**									
Tuberculosis	76 (74–86)	76 (74–79)	0	83 (79–85)	85 (82–89)	-2	76 (73–78)	76 (74–78)	0
Mumps	65 (63–67)	65 (63–68)	0	64 (62–67)	-		76 (71–78)	75 (71–79)	1
Seasonal influenza	-	-		-	-		-	68 (66–70)	
**Gastrointestinal or enteroviral diseases**									
Hepatitis A	71 (62–73)	72 (70–91)	-1	-	-		71 (68–73)	71 (69–73)	0
Hepatitis E	68 (66–70)	67 (63–70)	1	67 (65–69)	86 (71–91)	-19	68 (66–70)	72 (69–73)	-4
Typhoid and paratyphoid	-	72 (70–76)		72 (70–76)	72 (68–90)	0	76 (71–78)	75 (71–79)	1
Bacterial dysentery	-	-		-	86 (82–89)		70 (68–74)	71 (69–73)	-1
Acute hemorrhagic conjunctivitis	69 (67–71)	69 (67–71)	0	68 (66–70)	67 (65–71)	1	68 (67–70)	70 (69–72)	-2
Infectious diarrhea	88 (83–93)	86 (81–92)	2	-	87 (82–90)		-	70 (68–71)	
**Sexually transmitted or bloodborne diseases**									
Gonorrhea	68 (68–71)	65 (63–67)	3	60 (64–71)	60 (62–67)	0	67 (65–70)	64 (63–66)	3
Syphilis	72 (70–73)	-		-	-		-	-	
HIV/AIDS	76 (72–78)	65 (63–68)	11	67 (64–69)	65 (63–68)	2	76 (72–77)	65 (63–66)	11
Hepatitis B	65 (63–68)	71 (69–76)	-6	68 (67–69)	71 (69–74)	-3	72 (69–75)	72 (70–75)	0
Hepatitis C	67 (63–69)	72 (69–78)	-5	-	71 (69–73)		67 (66–69)	72 (69–76)	-5
**Vector-borne or zoonotic diseases**									
Typhus	70 (66–72)	71 (67–76)	-1	71 (67–74)	63 (62–66)	8	66 (62–70)	72 (68–75)	-6
Brucellosis	68 (67–70)	68 (67–70)	0	68 (66–69)	67 (65–68)	1	68 (67–69)	68 (66–69)	0

HFRS	68 (68–71)	68 (67–70)	0	68 (66–69)	70 (67–72)	-2	67 (63–68)	71 (68–77)	-4
Hydatid disease	74 (72–76)	73 (71–75）	1	76 (72–79)	75 (68–79)	1	73 (71–76)	73 (71–75)	0

"-"Indicates no inflection point and the age-specific incidence of infectious diseases exhibiting a linear increase with advancing age; Estimated point difference: Negative values indicate that the inflection points for the decline in age-specific incidence tend to occur earlier in males than females, and positive values indicate that the females than males. "0" indicates that the inflection point of decline is the same age for males and females. HFRS: hemorrhagic fever with renal syndrome.

Table S6. Continued.

Diseases	Estimated inflection points (95% CI)					
	Male			Female		
	2022	2014	Estimated point difference	2022	2014	Estimated point difference
**Respiratory diseases**						
Tuberculosis	69 (67–71)	76 (75–79)	-7	70 (68–72)	76 (75–78)	-6
Mumps	67 (64–71)	\	7	67 (64–70)	\	7
Seasonal influenza	68 (66–70)*	80 (74–84)	_	67 (64–69)*	\	
**Gastrointestinal or enteroviral diseases**						
Hepatitis A	71 (68–73)	_		70 (67–73)*	77 (72–89)	
Hepatitis E	68 (66–70)	76 (74–78)	-8	67 (65–69)	76 (70–84)	-9
Bacterial dysentery	67 (65–69)*	80 (71–86)		67 (66–69)*	80 (75–84)	
AHC	69 (67–72)	\	9	70 (67–72)	\	10
Infectious diarrhea	68 (66–70)*	84 (79–88)		68 (67–70)*	81 (78–85)	
**Sexually transmitted or bloodborne diseases**						
Gonorrhea	65 (63–69)	\	5	65 (63–70)	\	5
Syphilis	71 (68–73)	75 (72–77)	-4	70 (68–73)*	73 (69–78)	-3
HIV/AIDS	69 (66–71)	74 (72–76)	-5	68 (67–70)	\	8
Hepatitis B	69 (67–71)	\	9	69 (67–71)	76 (73–78)	-7
Hepatitis C	68 (66–70)	80 (74–85)	-12	68 (66–70)	76 (72–78)	-8
**Vector-borne or zoonotic diseases**						
Brucellosis	69 (68–71)	\	9	68 (67–69)	\	8
HFRS	\	70 (67–73)	-10	\	70 (67–73)	-10
Hydatid disease	74 (71–78)	77 (75–78)	-3	_	_	

Note: Of the 21 diseases examined, five (typhoid and paratyphoid, hand, foot and mouth disease, amoebic dysentery, typhus, and rabies) were excluded from the JPR analysis due to discontinuous age-specific incidence rates. "-" indicates that there was no inflection point of decline; "\" indicates that the incidence of the disease was highest at age 60 years and then declines with each passing year; "*" indicates that there was an upward inflection point and no downward inflection point; Estimated point difference: a negative value indicates that the inflection point of decline in the age-specific incidence tends to be earlier in 2022 than in 2014, and a positive value indicates that it will be later in 2022 than in 2014. HFRS: hemorrhagic fever with renal syndrome; AHC: acute hemorrhagic conjunctivitis.

Table S7. Comparison of crude average monthly incidences (1/100 000) of 21 NIDs between pre-COVID-19 pandemic and COVID-19 pandemic in China.

Disease	Pre-COVID-19	COVID-19 Stage I	COVID-19 Stage II	The percentage of change (%)	
				COVID-19 Stage I vs. Pre-COVID-19	COVID-19 Stage II vs. Pre-COVID-19
**Respiratory diseases**	**12·324**	**10·521**	**9·645**	**-14·63**	**-21·74**
Tuberculosis	10·126	7·919	7·503	-21·80	-25·90
Mumps	0·079	0·070	0·066	-11·39	-16·46
Seasonal influenza	2·119	2·532	2·076	19·49	-2·03
**Gastrointestinal or enteroviral diseases**	**6·158**	**3·957**	**5·943**	**-35·74**	**-3·49**
Hepatitis A	0·156	0·115	0·138	-26·28	-11·54
Hepatitis E	0·308	0·196	0·270	-36·36	-12·34
Hand, foot and mouth disease	0·007	0·000	0·004	-100·00	-42·86
Typhoid and paratyphoid	0·068	0·029	0·047	-57·35	-30·88
Bacterial dysentery	0·736	0·267	0·351	-63·72	-52·31
Acute hemorrhagic conjunctivitis	0·221	0·210	0·247	-4·98	11·76
Infectious diarrhea	4·656	3·138	4·883	-32·60	4·88
Amoebic dysentery	0·006	0·002	0·003	-66·67	-50·00
**Sexually transmitted or bloodborne diseases**	**14·740**	**13·468**	**14·968**	**-8·63**	**1·55**
Gonorrhea	0·204	0·087	0·143	-57·35	-29·90
Syphilis	4·514	4·322	4·819	-4·25	6·76
HIV/AIDS	0·973	0·899	1·012	-7·61	4·01
Hepatitis B	6·861	6·280	6·918	-8·47	0·83
Hepatitis C	2·188	1·880	2·076	-14·08	-5·12
**Vector-borne or zoonotic diseases**	**0·479**	**0·379**	**0·580**	**-20·88**	**21·09**

Typhus	0·010	0·004	0·013	-60·00	30·00
Brucellosis	0·342	0·312	0·471	-8·77	37·72
Hemorrhagic fever with renal syndrome	0·088	0·042	0·073	-52·27	-17·05
Rabies	0·008	0·002	0·003	-75·00	-62·50
Hydatid disease	0·031	0·019	0·020	-38·71	-35·48
**Total**	**33·702**	**28·325**	**31·135**	**-15·95**	**-7·62**

Supplemental Figures

Figure S1. Flow chart of the data processing procedure. NIDs: notifiable infectious diseases; CISDCP: China Information System for Disease Control and Prevention.

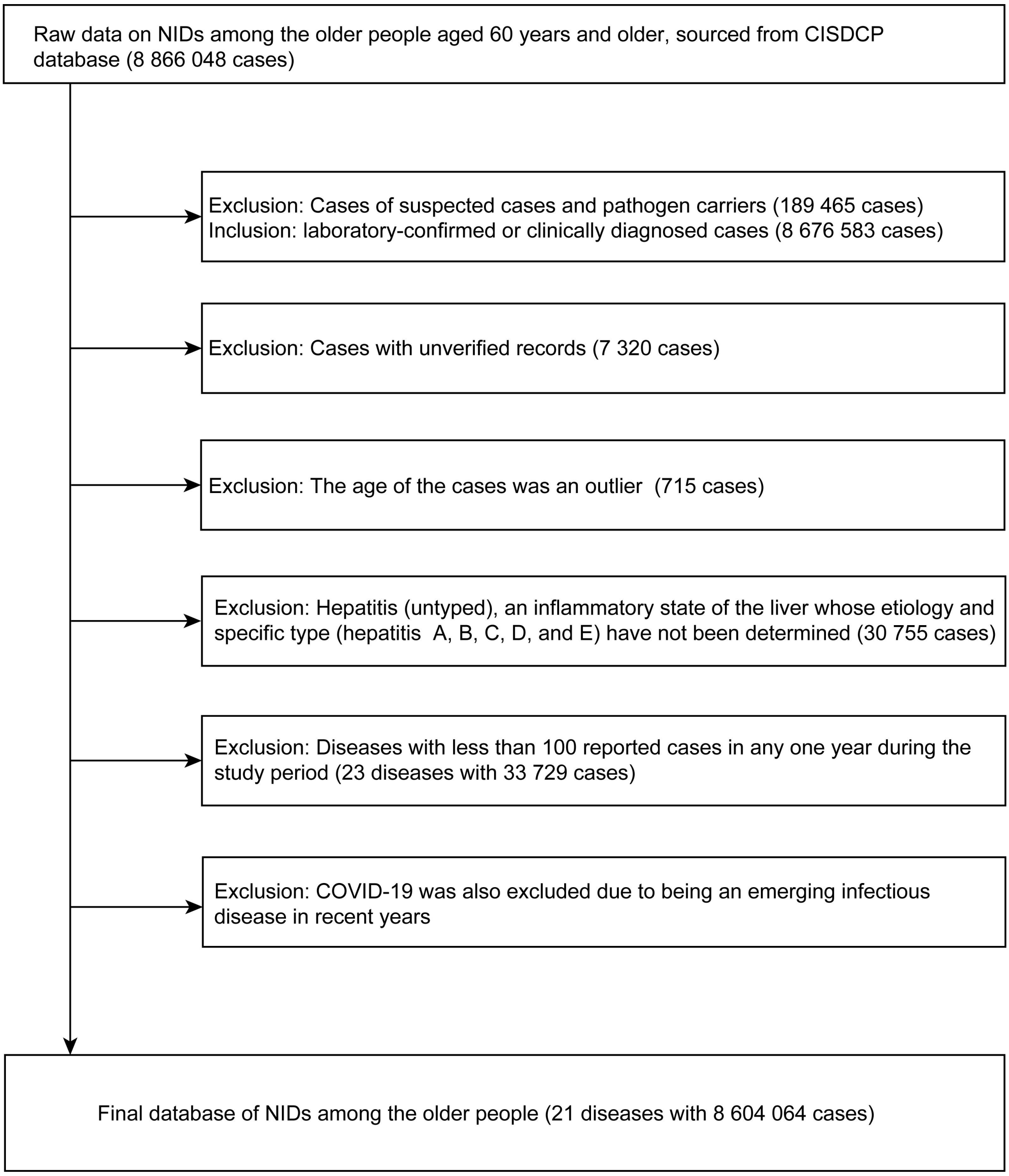

Figure S2. Ranking of 43 infectious diseases among different populations in China, 2014–2022. HFRS: hemorrhagic fever with renal syndrome; T/P: typhoid and paratyphoid; HFMD: hand, foot and mouth disease; AHC: acute hemorrhagic conjunctivitis; ECM: epidemic cerebrospinal meningitis; HIHPAI: human infection with highly pathogenic avian influenza. Note: Exclude COVID-19, poliomyelitis, and SARS.

Ranking	The entire population	Under 17 years of age	Younger adults	Older people
1	HFMD	HFMD	Hepatitis B	Tuberculosis
2	Infectious diarrhea	Seasonal influenza	Tuberculosis	Hepatitis B
3	Seasonal influenza	Infectious diarrhea	Syphilis	Infectious diarrhea
4	Hepatitis B	Mumps	Infectious diarrhea	Syphilis
5	Tuberculosis	Scarlet fever	Seasonal influenza	Hepatitis C
6	Syphilis	Bacterial dysentery	Hepatitis C	Seasonal influenza
7	Hepatitis C	Tuberculosis	HIV/AIDS	HIV/AIDS
8	Mumps	Pertussis	Gonorrhea	Bacterial dysentery
9	HIV/AIDS	Hepatitis B	Brucellosis	Brucellosis
10	Gonorrhea	Syphilis	Bacterial dysentery	Hepatitis E
11	Bacterial dysentery	AHC	Mumps	AHC
12	Scarlet fever	Measles	AHC	Gonorrhea
13	Brucellosis	Gonorrhea	Hepatitis E	Hepatitis A
14	AHC	Rubella	Hepatitis (untyped)	Hepatitis (untyped)
15	Hepatitis E	T/P	Hepatitis A	HFRS
16	Hepatitis A	Hepatitis A	HFMD	Mumps
17	Hepatitis (untyped)	HIV/AIDS	Dengue	T/P
18	Measles	Brucellosis	HFRS	Dengue
19	Pertussis	Hepatitis C	Measles	Schistosomiasis
20	Dengue	Hepatitis (untyped)	T/P	Hydatid disease
21	T/P	Dengue	Schistosomiasis	Typhus
22	HFRS	Amoebic dysentery	Rubella	Rabies
23	Rubella	HFRS	Hydatid disease	HFMD
24	Schistosomiasis	Japanese encephalitis	Malaria	Japanese encephalitis
25	Hydatid disease	Hydatid disease	Typhus	Amoebic dysentery
26	Malaria	Typhus	Scarlet fever	Leptospirosis
27	Typhus	Hepatitis E	Amoebic dysentery	Leprosy
28	Amoebic dysentery	Kala-azar	Leprosy	Hepatitis D
29	Japanese encephalitis	Neonatal tetanus	Anthrax	H7N9
30	Rabies	ECM	Japanese encephalitis	Rubella
31	Leprosy	Rabies	Rabies	Malaria
32	Anthrax	Schistosomiasis	Leptospirosis	Measles
33	Leptospirosis	Malaria	Hepatitis D	Anthrax
34	Kala-azar	Leprosy	Pertussis	Pertussis
35	Hepatitis D	Anthrax	Kala-azar	Kala-azar
36	Neonatal tetanus	Leptospirosis	H7N9	Scarlet fever
37	H7N9	H7N9	ECM	Cholera
38	ECM	Hepatitis D	Cholera	ECM
39	Cholera	Cholera	Plague	Plague
40	Plague	HIHPAI	HIHPAI	HIHPAI
41	HIHPAI	Diphtheria	Diphtheria	Diphtheria
42	Diphtheria	Plague		
43	Filariasis			

Figure S3. Geographical distribution of seven ecological zones in the mainland of China. GEDs: gastrointestinal or enteroviral diseases; STBDs: sexually transmitted or bloodborne diseases; VBZDs: vector-borne or zoonotic diseases.

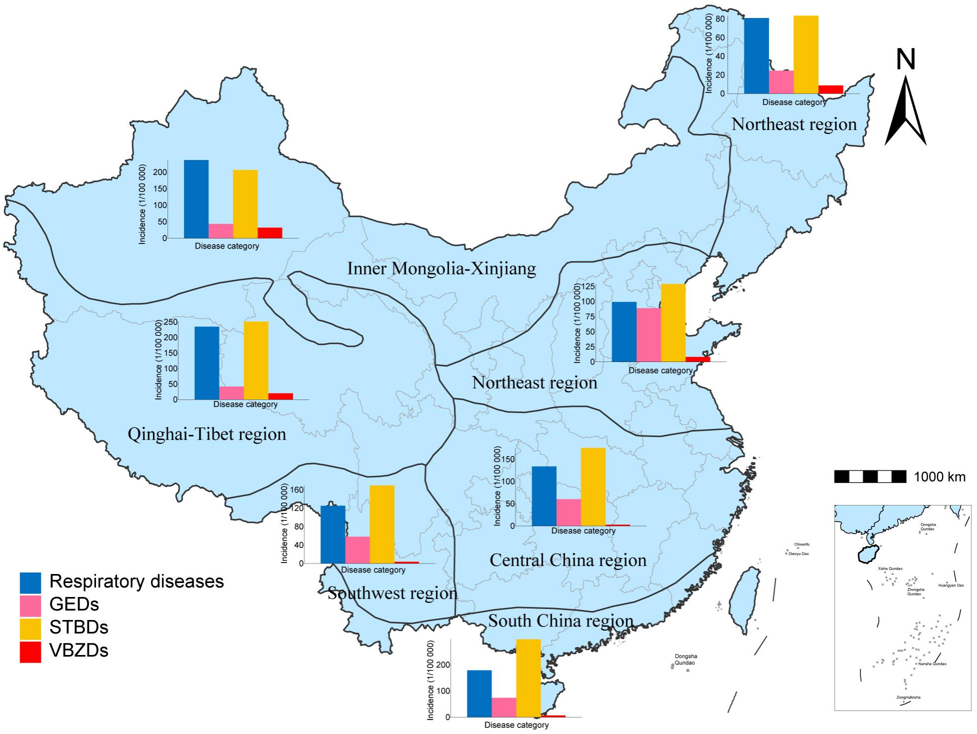

Figure S4. Overall temporal trends in incidences of four categories of infectious diseases among the older people in China, 2014–2022. GEDs: gastrointestinal or enteroviral diseases; STBDs: sexually transmitted or bloodborne diseases; VBZDs: vector-borne or zoonotic diseases.

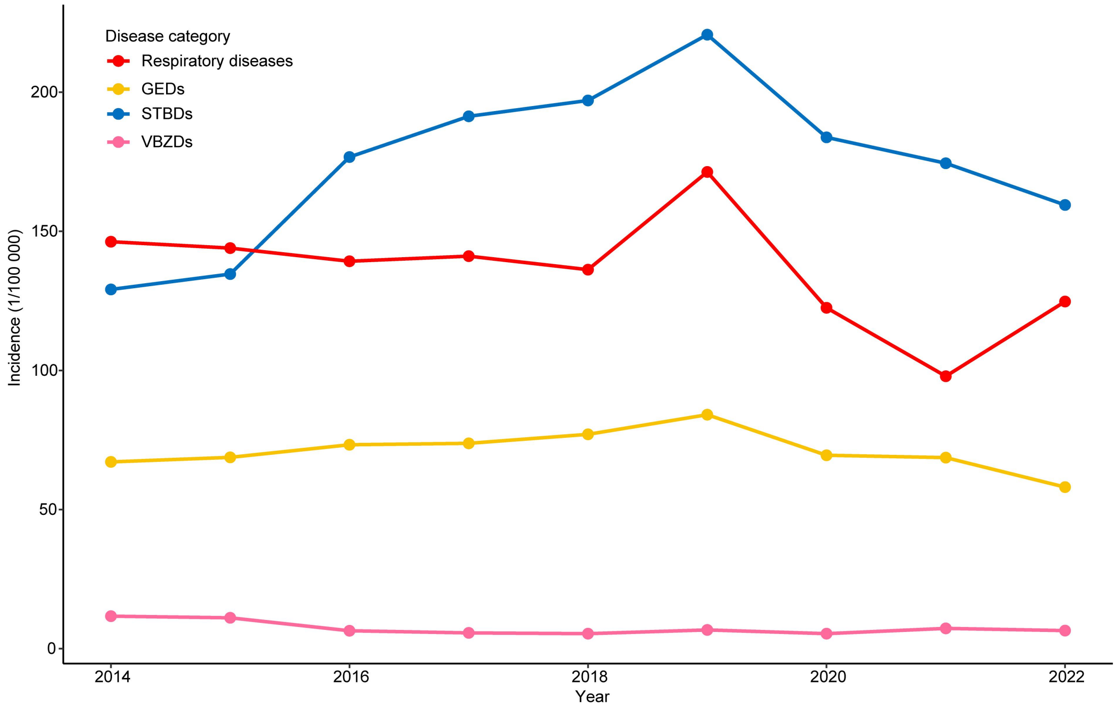

Figure S5. Overall temporal trends in incidences of 21 NIDs among the entire population in China, 2014–2022. a. The entire population. b. Male. c. Female. d. Under 17 years of age. e. Younger adults. f. Older people. AHC: acute hemorrhagic conjunctivitis; T/P: typhoid and paratyphoid; HFMD: hand, foot and mouth disease; HFRS: hemorrhagic fever with renal syndrome.

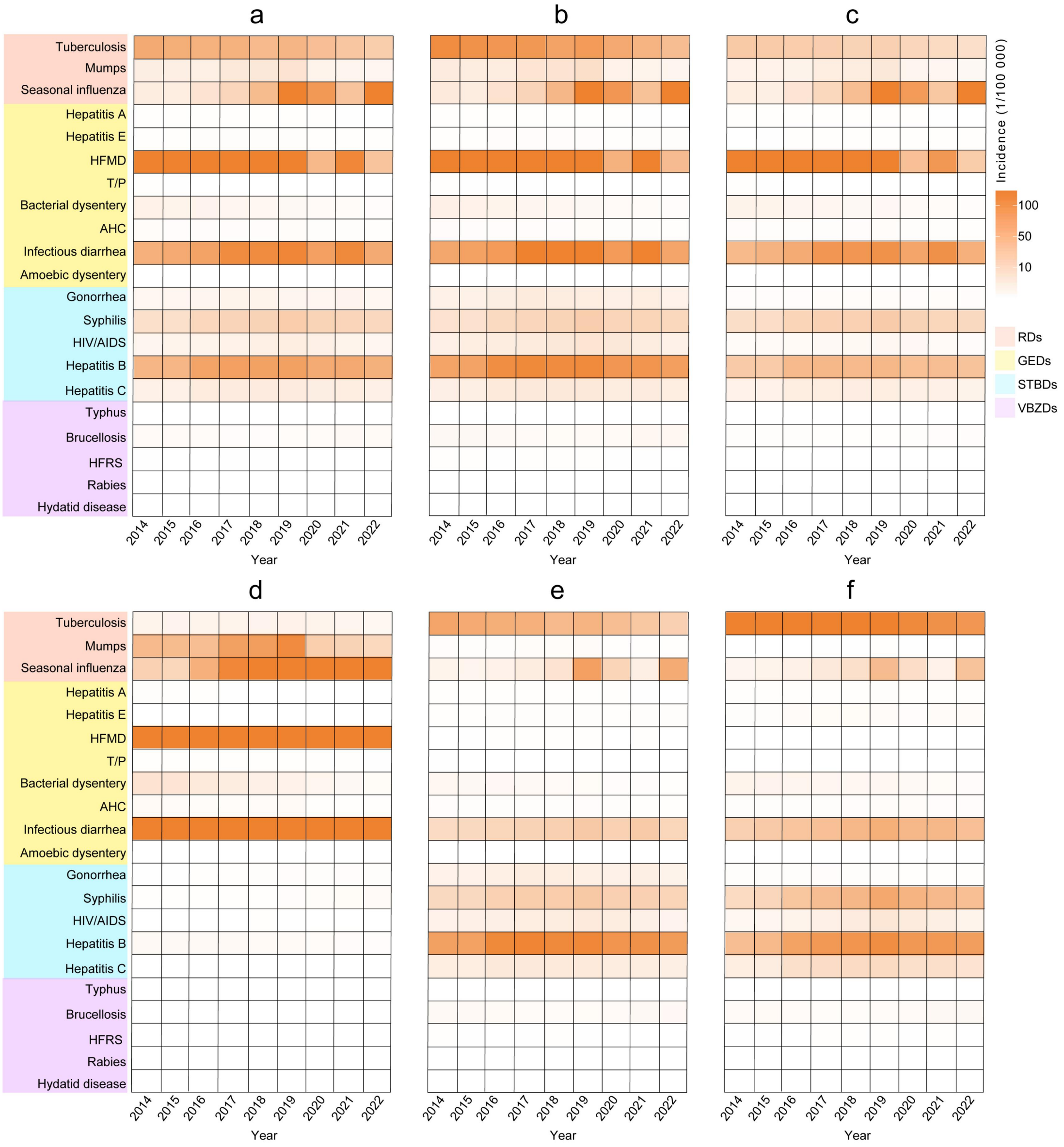

Figure S6. Seasonal distribution of the incidences for 21 NIDs among the older people in China, 2014–2022. a. Respiratory diseases. b. Gastrointestinal or enteroviral diseases. c. Sexually transmitted or bloodborne diseases. d. Vector-borne or zoonotic diseases. AHC: acute hemorrhagic conjunctivitis; T/P: typhoid and paratyphoid; HFMD: hand, foot and mouth disease; HFRS: hemorrhagic fever with renal syndrome.

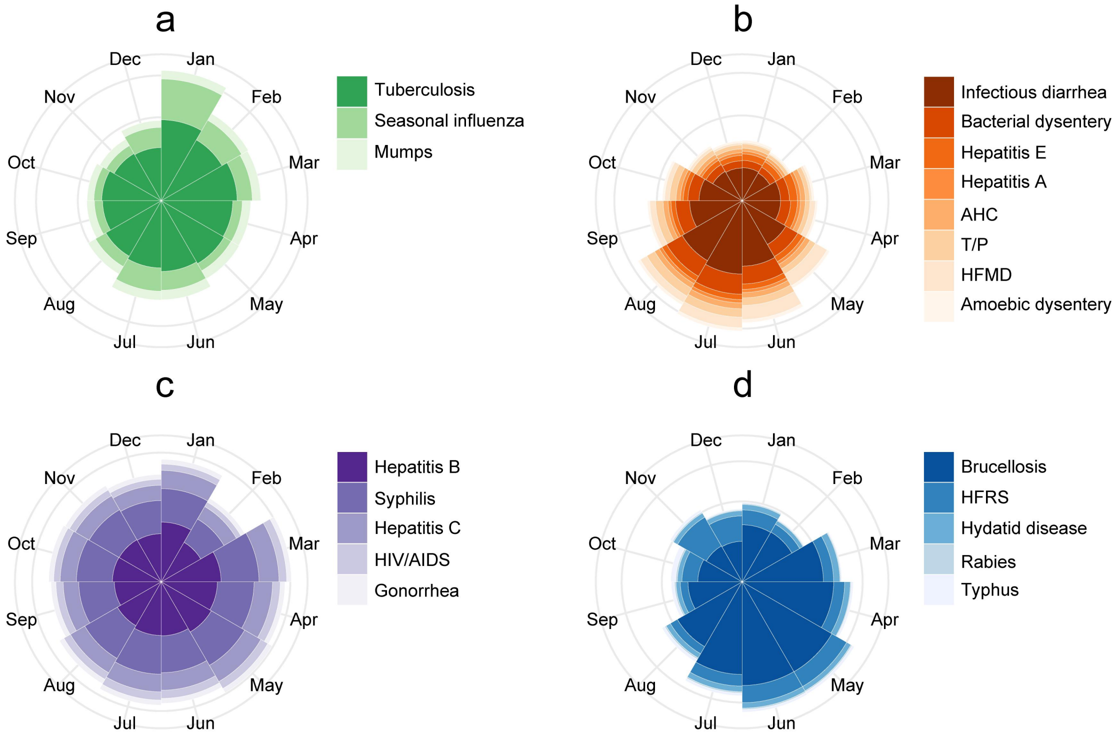

Figure S7. Ranking of incidence of each of the 21 NIDs by age group, from 2014 to 2022. TB: tuberculosis; Flu: seasonal influenza; HFMD: hand, foot and mouth disease; T/P: typhoid and paratyphoid; BD: bacterial dysentery; AHC: acute hemorrhagic conjunctivitis; ID: infectious diarrhea other than cholera, dysentery, typhoid and paratyphoid; AD: amoebic dysentery; HFRS: hemorrhagic fever with renal syndrome.

Ranking/ Age group	1	2	3	4	5	6	7	8	9	10	11	12	13	14	15	16	17	18	19	20	21
a)Composition of the pathogen spectrum in 2014																					
60–69	TB	Hepatitis B	ID	Syphilis	Hepatitis C	BD	Flu	HIV/AIDS	Brucellosis	Gonorrhea	Hepatitis E	AHC	Hepatitis A	HFRS	Mumps	T/P	Hydatid disease	Typhus	HFMD	AD	Rabies
70–79	TB	Hepatitis B	ID	Syphilis	Hepatitis C	BD	Flu	HIV/AIDS	Hepatitis E	Brucellosis	AHC	Hepatitis A	Gonorrhea	T/P	HFRS	Mumps	Hydatid disease	Rabies	Typhus	HFMD	AD
80–89	TB	ID	Syphilis	Hepatitis B	Hepatitis C	BD	Flu	HIV/AIDS	Hepatitis E	AHC	Hepatitis A	Gonorrhea	Brucellosis	T/P	Mumps	HFRS	Rabies	Hydatid disease	AD	HFMD	Typhus
≥90	TB	ID	Syphilis	Hepatitis B	BD	Hepatitis C	Flu	Hepatitis E	Hepatitis A	AHC	Gonorrhea	T/P	HIV/AIDS	Mumps	Brucellosis	HFRS	Rabies	Hydatid disease	AD	HFMD	
b)Composition of the pathogen spectrum in 2015																					
60–69	TB	Hepatitis B	ID	Syphilis	Hepatitis C	Flu	BD	HIV/AIDS	Brucellosis	Gonorrhea	Hepatitis E	AHC	Hepatitis A	HFRS	Mumps	T/P	Hydatid disease	Typhus	Rabies	HFMD	AD
70–79	TB	Hepatitis B	ID	Syphilis	Hepatitis C	BD	Flu	HIV/AIDS	Brucellosis	Hepatitis E	AHC	Hepatitis A	Gonorrhea	T/P	HFRS	Mumps	Hydatid disease	Rabies	Typhus	AD	HFMD
80–89	TB	ID	Syphilis	Hepatitis B	Hepatitis C	BD	Flu	HIV/AIDS	Hepatitis E	Hepatitis A	AHC	Brucellosis	T/P	Gonorrhea	Mumps	HFRS	Hydatid disease	Rabies	AD	HFMD	Typhus
≥90	TB	ID	Syphilis	Hepatitis B	BD	Hepatitis C	Flu	HIV/AIDS	Hepatitis E	AHC	Hepatitis A	T/P	Gonorrhea	Mumps	HFRS	Brucellosis	Typhus	Rabies	Hydatid disease		
c)Composition of the pathogen spectrum in 2016																					
60–69	TB	Hepatitis B	ID	Syphilis	Hepatitis C	Flu	HIV/AIDS	BD	Brucellosis	Hepatitis E	Gonorrhea	AHC	Hepatitis A	HFRS	Mumps	T/P	Hydatid disease	Typhus	Rabies	HFMD	AD
70–79	TB	Hepatitis B	Syphilis	ID	Hepatitis C	Flu	BD	HIV/AIDS	Hepatitis E	AHC	Brucellosis	Hepatitis A	Gonorrhea	T/P	Mumps	HFRS	Hydatid disease	Rabies	Typhus	HFMD	AD
80–89	TB	Syphilis	ID	Hepatitis B	Hepatitis C	Flu	BD	HIV/AIDS	Hepatitis E	Hepatitis A	AHC	Gonorrhea	T/P	Brucellosis	Mumps	HFRS	Hydatid disease	AD	HFMD	Rabies	Typhus
≥90	Syphilis	ID	TB	Hepatitis B	Hepatitis C	Flu	BD	Hepatitis E	Hepatitis A	HIV/AIDS	AHC	Gonorrhea	T/P	Mumps	Brucellosis	AD	HFRS	Rabies	Hydatid disease	Typhus	
d)Composition of the pathogen spectrum in 2017																					
60–69	TB	Hepatitis B	ID	Syphilis	Hepatitis C	Flu	HIV/AIDS	BD	Brucellosis	Hepatitis E	Gonorrhea	AHC	Hepatitis A	HFRS	Mumps	T/P	Hydatid disease	Rabies	Typhus	HFMD	AD
70–79	TB	Hepatitis B	Syphilis	ID	Hepatitis C	Flu	HIV/AIDS	BD	Hepatitis E	AHC	Hepatitis A	Brucellosis	Gonorrhea	T/P	HFRS	Mumps	Hydatid disease	Typhus	Rabies	AD	HFMD
80–89	TB	Syphilis	ID	Hepatitis B	Hepatitis C	Flu	BD	HIV/AIDS	Hepatitis E	Hepatitis A	AHC	T/P	Gonorrhea	Mumps	Brucellosis	HFRS	Hydatid disease	AD	Typhus	HFMD	Rabies
≥90	Syphilis	ID	TB	Hepatitis B	Flu	Hepatitis C	BD	Hepatitis E	Hepatitis A	HIV/AIDS	AHC	T/P	Gonorrhea	Mumps	HFRS	Brucellosis	HFMD	Hydatid disease	Typhus	Rabies	AD
e)Composition of the pathogen spectrum in 2018																					
60–69	Hepatitis B	TB	ID	Syphilis	Hepatitis C	Flu	HIV/AIDS	BD	Brucellosis	Hepatitis E	Gonorrhea	AHC	Hepatitis A	HFRS	Mumps	T/P	Hydatid disease	Typhus	HFMD	Rabies	AD
70–79	TB	Hepatitis B	Syphilis	ID	Hepatitis C	Flu	HIV/AIDS	BD	Hepatitis E	AHC	Hepatitis A	Brucellosis	Gonorrhea	HFRS	T/P	Mumps	Hydatid disease	Typhus	AD	Rabies	HFMD
80–89	TB	Syphilis	ID	Hepatitis B	Flu	Hepatitis C	BD	HIV/AIDS	Hepatitis E	AHC	Hepatitis A	T/P	Gonorrhea	Mumps	Brucellosis	HFRS	Hydatid disease	Typhus	AD	Rabies	HFMD
≥90	Syphilis	ID	TB	Flu	Hepatitis B	Hepatitis C	BD	HIV/AIDS	Hepatitis E	Hepatitis A	AHC	T/P	Gonorrhea	Mumps	HFRS	Brucellosis	Hydatid disease	AD	HFMD		
f)Composition of the pathogen spectrum in 2019																					
60–69	Hepatitis B	TB	ID	Syphilis	Flu	Hepatitis C	HIV/AIDS	BD	Brucellosis	Hepatitis E	AHC	Gonorrhea	Hepatitis A	Mumps	HFRS	T/P	Hydatid disease	Typhus	HFMD	Rabies	AD
70–79	TB	Hepatitis B	Syphilis	ID	Flu	Hepatitis C	HIV/AIDS	BD	Hepatitis E	AHC	Hepatitis A	Brucellosis	Gonorrhea	Mumps	HFRS	T/P	Hydatid disease	Typhus	Rabies	HFMD	AD
80–89	TB	Syphilis	ID	Flu	Hepatitis B	Hepatitis C	HIV/AIDS	BD	Hepatitis E	Hepatitis A	AHC	T/P	Mumps	Gonorrhea	Brucellosis	HFRS	Hydatid disease	AD	Typhus	HFMD	Rabies
≥90	Syphilis	ID	Flu	TB	Hepatitis B	Hepatitis C	BD	HIV/AIDS	Hepatitis A	Hepatitis E	AHC	T/P	Mumps	Gonorrhea	HFRS	Brucellosis	AD	Hydatid disease	Typhus	HFMD	Rabies
g)Composition of the pathogen spectrum in 2020																					
60–69	Hepatitis B	TB	ID	Syphilis	Hepatitis C	Flu	HIV/AIDS	Brucellosis	BD	AHC	Hepatitis E	Gonorrhea	Hepatitis A	HFRS	Mumps	T/P	Hydatid disease	Typhus	HFMD	AD	Rabies
70–79	TB	Hepatitis B	Syphilis	ID	Flu	Hepatitis C	HIV/AIDS	BD	AHC	Brucellosis	Hepatitis E	Hepatitis A	Gonorrhea	HFRS	Mumps	T/P	Hydatid disease	Typhus	AD	Rabies	HFMD
80–89	TB	Syphilis	ID	Hepatitis B	Flu	Hepatitis C	HIV/AIDS	BD	AHC	Hepatitis E	Hepatitis A	T/P	Brucellosis	Mumps	Gonorrhea	HFRS	Hydatid disease	Typhus	AD	HFMD	Rabies
≥90	Syphilis	ID	TB	Flu	Hepatitis B	Hepatitis C	BD	HIV/AIDS	AHC	Hepatitis A	Hepatitis E	Mumps	T/P	Gonorrhea	Brucellosis	HFRS	Hydatid disease	HFMD	Rabies		
h)Composition of the pathogen spectrum in 2021																					
60–69	Hepatitis B	TB	ID	Syphilis	Hepatitis C	HIV/AIDS	Flu	Brucellosis	BD	Hepatitis E	AHC	Gonorrhea	Hepatitis A	HFRS	Mumps	T/P	Hydatid disease	Typhus	HFMD	Rabies	AD
70–79	TB	Hepatitis B	Syphilis	ID	Hepatitis C	Flu	HIV/AIDS	BD	Brucellosis	Hepatitis E	AHC	Hepatitis A	Gonorrhea	HFRS	Mumps	T/P	Hydatid disease	Typhus	AD	Rabies	HFMD
80–89	TB	Syphilis	ID	Hepatitis B	Hepatitis C	Flu	HIV/AIDS	BD	Hepatitis E	AHC	Hepatitis A	Brucellosis	T/P	Mumps	Gonorrhea	HFRS	Hydatid disease	Typhus	AD	HFMD	
≥90	Syphilis	ID	TB	Hepatitis B	Flu	Hepatitis C	BD	Hepatitis E	HIV/AIDS	Hepatitis A	AHC	T/P	Mumps	Gonorrhea	Brucellosis	HFRS	Hydatid disease	Typhus	AD	HFMD	
i)Composition of the pathogen spectrum in 2022																					
60–69	Hepatitis B	TB	ID	Syphilis	Flu	Hepatitis C	HIV/AIDS	Brucellosis	Hepatitis E	BD	AHC	Gonorrhea	Hepatitis A	Mumps	HFRS	T/P	Hydatid disease	Typhus	HFMD	Rabies	AD
70–79	TB	Hepatitis B	Syphilis	ID	Flu	Hepatitis C	HIV/AIDS	Brucellosis	BD	Hepatitis E	AHC	Hepatitis A	Gonorrhea	HFRS	Mumps	T/P	Hydatid disease	Typhus	Rabies	AD	HFMD
80–89	TB	Syphilis	Flu	ID	Hepatitis B	Hepatitis C	HIV/AIDS	BD	Hepatitis E	AHC	Hepatitis A	Brucellosis	T/P	Mumps	Gonorrhea	HFRS	Hydatid disease	Typhus	AD	HFMD	Rabies
≥90	Flu	Syphilis	ID	TB	Hepatitis B	Hepatitis C	BD	HIV/AIDS	Hepatitis E	AHC	Hepatitis A	T/P	Brucellosis	Mumps	Gonorrhea	Hydatid disease	HFRS	HFMD	Typhus	AD	

Figure S8. Infectious diseases with greatest incidence by age, 2014–2022. a. Leading disease in incidence of respiratory diseases. b. Leading disease in incidence of gastrointestinal or enteroviral diseases. c. Leading disease in incidence of sexually transmitted or bloodborne diseases. d. Leading disease in incidence of vector-borne or zoonotic diseases. TB: tuberculosis; Flu: seasonal influenza; ID: infectious diarrhea other than cholera, dysentery, typhoid and paratyphoid; HFRS: hemorrhagic fever with renal syndrome.

a (columns 2014–2022, first group); b (columns 2014–2022, second group)

Year/age	2014	2015	2016	2017	2018	2019	2020	2021	2022	2014	2015	2016	2017	2018	2019	2020	2021	2022
60	TB	TB	TB	TB	TB	TB	TB	TB	TB	ID	ID	ID	ID	ID	ID	ID	ID	ID
61	TB	TB	TB	TB	TB	TB	TB	TB	TB	ID	ID	ID	ID	ID	ID	ID	ID	ID
62	TB	TB	TB	TB	TB	TB	TB	TB	TB	ID	ID	ID	ID	ID	ID	ID	ID	ID
63	TB	TB	TB	TB	TB	TB	TB	TB	TB	ID	ID	ID	ID	ID	ID	ID	ID	ID
64	TB	TB	TB	TB	TB	TB	TB	TB	TB	ID	ID	ID	ID	ID	ID	ID	ID	ID
65	TB	TB	TB	TB	TB	TB	TB	TB	TB	ID	ID	ID	ID	ID	ID	ID	ID	ID
66	TB	TB	TB	TB	TB	TB	TB	TB	TB	ID	ID	ID	ID	ID	ID	ID	ID	ID
67	TB	TB	TB	TB	TB	TB	TB	TB	TB	ID	ID	ID	ID	ID	ID	ID	ID	ID
68	TB	TB	TB	TB	TB	TB	TB	TB	TB	ID	ID	ID	ID	ID	ID	ID	ID	ID
69	TB	TB	TB	TB	TB	TB	TB	TB	TB	ID	ID	ID	ID	ID	ID	ID	ID	ID
70	TB	TB	TB	TB	TB	TB	TB	TB	TB	ID	ID	ID	ID	ID	ID	ID	ID	ID
71	TB	TB	TB	TB	TB	TB	TB	TB	TB	ID	ID	ID	ID	ID	ID	ID	ID	ID
72	TB	TB	TB	TB	TB	TB	TB	TB	TB	ID	ID	ID	ID	ID	ID	ID	ID	ID
73	TB	TB	TB	TB	TB	TB	TB	TB	TB	ID	ID	ID	ID	ID	ID	ID	ID	ID
74	TB	TB	TB	TB	TB	TB	TB	TB	TB	ID	ID	ID	ID	ID	ID	ID	ID	ID
75	TB	TB	TB	TB	TB	TB	TB	TB	TB	ID	ID	ID	ID	ID	ID	ID	ID	ID
76	TB	TB	TB	TB	TB	TB	TB	TB	TB	ID	ID	ID	ID	ID	ID	ID	ID	ID
77	TB	TB	TB	TB	TB	TB	TB	TB	TB	ID	ID	ID	ID	ID	ID	ID	ID	ID
78	TB	TB	TB	TB	TB	TB	TB	TB	TB	ID	ID	ID	ID	ID	ID	ID	ID	ID
79	TB	TB	TB	TB	TB	TB	TB	TB	TB	ID	ID	ID	ID	ID	ID	ID	ID	ID
80	TB	TB	TB	TB	TB	TB	TB	TB	TB	ID	ID	ID	ID	ID	ID	ID	ID	ID
81	TB	TB	TB	TB	TB	TB	TB	TB	TB	ID	ID	ID	ID	ID	ID	ID	ID	ID
82	TB	TB	TB	TB	TB	TB	TB	TB	TB	ID	ID	ID	ID	ID	ID	ID	ID	ID
83	TB	TB	TB	TB	TB	TB	TB	TB	TB	ID	ID	ID	ID	ID	ID	ID	ID	ID
84	TB	TB	TB	TB	TB	TB	TB	TB	TB	ID	ID	ID	ID	ID	ID	ID	ID	ID
85	TB	TB	TB	TB	TB	TB	TB	TB	TB	ID	ID	ID	ID	ID	ID	ID	ID	ID
86	TB	TB	TB	TB	TB	TB	TB	TB	TB	ID	ID	ID	ID	ID	ID	ID	ID	ID
87	TB	TB	TB	TB	TB	TB	TB	TB	TB	ID	ID	ID	ID	ID	ID	ID	ID	ID
88	TB	TB	TB	TB	TB	TB	TB	TB	TB	ID	ID	ID	ID	ID	ID	ID	ID	ID
89	TB	TB	TB	TB	TB	TB	TB	TB	Flu	ID	ID	ID	ID	ID	ID	ID	ID	ID
90	TB	TB	TB	TB	TB	Flu	TB	TB	Flu	ID	ID	ID	ID	ID	ID	ID	ID	ID
91	TB	TB	TB	TB	TB	Flu	TB	TB	Flu	ID	ID	ID	ID	ID	ID	ID	ID	ID
92	TB	TB	TB	TB	TB	Flu	TB	TB	Flu	ID	ID	ID	ID	ID	ID	ID	ID	ID
93	TB	TB	TB	TB	TB	Flu	TB	TB	Flu	ID	ID	ID	ID	ID	ID	ID	ID	ID
94	TB	TB	TB	TB	TB	Flu	Flu TB	TB	Flu	ID	ID	ID	ID	ID	ID	ID	ID	ID
95	TB	TB	TB	TB	TB	Flu	TB	TB	Flu	ID	ID	ID	ID	ID	ID	ID	ID	ID
96	TB	TB	TB	TB	TB	Flu	TB	TB	Flu	ID	ID	ID	ID	ID	ID	ID	ID	ID
97	TB	TB	TB	TB	TB	Flu	TB	TB	Flu	ID	ID	ID	ID	ID	ID	ID	ID	ID
98	TB	TB	TB	TB	TB	Flu	Flu TB	TB	Flu	ID	ID	ID	ID	ID	ID	ID	ID	ID
99	TB	TB	TB	TB	TB	TB	TB	TB	Flu	ID	ID	ID	ID	ID	ID	ID	ID	ID
≥100	TB	Flu TB	TB	TB	Flu TB	TB	TB	TB	Flu	ID	ID	ID	ID	ID	ID	ID	ID	ID

c (columns 2014–2022, first group); d (columns 2014–2022, second group)

Year/age	2014	2015	2016	2017	2018	2019	2020	2021	2022	2014	2015	2016	2017	2018	2019	2020	2021	2022
60	Hepatitis B	Hepatitis B	Hepatitis B	Hepatitis B	Hepatitis B	Hepatitis B	Hepatitis B	Hepatitis B	Hepatitis B	Brucellosis	Brucellosis	Brucellosis	Brucellosis	Brucellosis	Brucellosis	Brucellosis	Brucellosis	Brucellosis
61	Hepatitis B	IHepatitis B	Hepatitis B	Hepatitis B	Hepatitis B	Hepatitis B	Hepatitis B	Hepatitis B	Hepatitis B	Brucellosis	Brucellosis	Brucellosis	Brucellosis	Brucellosis	Brucellosis	Brucellosis	Brucellosis	Brucellosis
62	Hepatitis B	Hepatitis B	Hepatitis B	Hepatitis B	Hepatitis B	Hepatitis B	Hepatitis B	Hepatitis B	Hepatitis B	Brucellosis	Brucellosis	Brucellosis	Brucellosis	Brucellosis	Brucellosis	Brucellosis	Brucellosis	Brucellosis
63	Hepatitis B	Hepatitis B	Hepatitis B	Hepatitis B	Hepatitis B	Hepatitis B	Hepatitis B	Hepatitis B	Hepatitis B	Brucellosis	Brucellosis	Brucellosis	Brucellosis	Brucellosis	Brucellosis	Brucellosis	Brucellosis	Brucellosis
64	Hepatitis B	Hepatitis B	Hepatitis B	Hepatitis B	Hepatitis B	Hepatitis B	Hepatitis B	Hepatitis B	Hepatitis B	Brucellosis	Brucellosis	Brucellosis	Brucellosis	Brucellosis	Brucellosis	Brucellosis	Brucellosis	Brucellosis
65	Hepatitis B	Hepatitis B	Hepatitis B	Hepatitis B	Hepatitis B	Hepatitis B	Hepatitis B	Hepatitis B	Hepatitis B	Brucellosis	Brucellosis	Brucellosis	Brucellosis	Brucellosis	Brucellosis	Brucellosis	Brucellosis	Brucellosis
66	Hepatitis B	Hepatitis B	Hepatitis B	Hepatitis B	Hepatitis B	Hepatitis B	Hepatitis B	Hepatitis B	Hepatitis B	Brucellosis	Brucellosis	Brucellosis	Brucellosis	Brucellosis	Brucellosis	Brucellosis	Brucellosis	Brucellosis
67	Hepatitis B	Hepatitis B	Hepatitis B	Hepatitis B	Hepatitis B	Hepatitis B	Hepatitis B	Hepatitis B	Hepatitis B	Brucellosis	Brucellosis	Brucellosis	Brucellosis	Brucellosis	Brucellosis	Brucellosis	Brucellosis	Brucellosis
68	Hepatitis B	Hepatitis B	Hepatitis B	Hepatitis B	Hepatitis B	Hepatitis B	Hepatitis B	Hepatitis B	Hepatitis B	Brucellosis	Brucellosis	Brucellosis	Brucellosis	Brucellosis	Brucellosis	Brucellosis	Brucellosis	Brucellosis
69	Hepatitis B	Hepatitis B	Hepatitis B	Hepatitis B	Hepatitis B	Hepatitis B	Hepatitis B	Hepatitis B	Hepatitis B	Brucellosis	Brucellosis	Brucellosis	Brucellosis	Brucellosis	Brucellosis	Brucellosis	Brucellosis	Brucellosis
70	Hepatitis B	Hepatitis B	Hepatitis B	Hepatitis B	Hepatitis B	Hepatitis B	Hepatitis B	Hepatitis B	Hepatitis B	Brucellosis	Brucellosis	Brucellosis	Brucellosis	Brucellosis	Brucellosis	Brucellosis	Brucellosis	Brucellosis
71	Hepatitis B	Hepatitis B	Hepatitis B	Hepatitis B	Hepatitis B	Hepatitis B	Hepatitis B	Hepatitis B	Hepatitis B	Brucellosis	Brucellosis	Brucellosis	Brucellosis	Brucellosis	Brucellosis	Brucellosis	Brucellosis	Brucellosis
72	Hepatitis B	Hepatitis B	Hepatitis B	Hepatitis B	Hepatitis B	Hepatitis B	Hepatitis B	Hepatitis B	Hepatitis B	Brucellosis	Brucellosis	Brucellosis	Brucellosis	Brucellosis	Brucellosis	Brucellosis	Brucellosis	Brucellosis
73	Hepatitis B	Hepatitis B	Hepatitis B	Hepatitis B	Hepatitis B	Hepatitis B	Hepatitis B	Hepatitis B	Hepatitis B	Brucellosis	Brucellosis	Brucellosis	Brucellosis	Brucellosis	Brucellosis	Brucellosis	Brucellosis	Brucellosis
74	Hepatitis B	Hepatitis B	Hepatitis B	Hepatitis B	Hepatitis B	Hepatitis B	Hepatitis B	Hepatitis B	Hepatitis B	Brucellosis	Brucellosis	Brucellosis	Brucellosis	Brucellosis	Brucellosis	Brucellosis	Brucellosis	Brucellosis
75	Hepatitis B	Hepatitis B	Hepatitis B	IHepatitis B	Hepatitis B	Syphilis	Hepatitis B	Hepatitis B	Hepatitis B	Brucellosis	Brucellosis	Brucellosis	Brucellosis	Brucellosis	Brucellosis	Brucellosis	Brucellosis	Brucellosis
76	Hepatitis B	Hepatitis B	Hepatitis B	Hepatitis B	Syphilis	Syphilis	Syphilis	Hepatitis B	Hepatitis B	Brucellosis	Brucellosis	Brucellosis	Brucellosis	Brucellosis	Brucellosis	Brucellosis	Brucellosis	Brucellosis
77	Hepatitis B	Hepatitis B	Hepatitis B	Syphilis	Syphilis	Syphilis	Syphilis	Syphilis	Hepatitis B	Brucellosis	Brucellosis	Brucellosis	Brucellosis	Brucellosis	Brucellosis	Brucellosis	Brucellosis	Brucellosis
78	Hepatitis B	Hepatitis B	Syphilis	Syphilis	Syphilis	Syphilis	Syphilis	Syphilis	Syphilis	Brucellosis	Brucellosis	Brucellosis	Brucellosis	Brucellosis	Brucellosis	Brucellosis	Brucellosis	Brucellosis
79	Hepatitis B	Syphilis	Syphilis	Syphilis	Syphilis	Syphilis	Syphilis	Syphilis	Syphilis	Brucellosis	Brucellosis	Brucellosis	Brucellosis	Brucellosis	Brucellosis	Brucellosis	Brucellosis	Brucellosis
80	Hepatitis B	Syphilis	Syphilis	Syphilis	Syphilis	Syphilis	Syphilis	Syphilis	Syphilis	Brucellosis	Brucellosis	Brucellosis	Brucellosis	Brucellosis	Brucellosis	Brucellosis	Brucellosis	Brucellosis
81	Syphilis	Syphilis	Syphilis	Syphilis	Syphilis	Syphilis	Syphilis	Syphilis	Syphilis	Brucellosis	Brucellosis	Brucellosis	Brucellosis	Brucellosis	Brucellosis	Brucellosis	Brucellosis	Brucellosis
82	Syphilis	Syphilis	Syphilis	Syphilis	Syphilis	Syphilis	Syphilis	Syphilis	Syphilis	Brucellosis	Brucellosis	Brucellosis	Brucellosis	Brucellosis	Brucellosis	Brucellosis	Brucellosis	Brucellosis
83	Syphilis	Syphilis	Syphilis	Syphilis	Syphilis	Syphilis	Syphilis	Syphilis	Syphilis	Brucellosis	Brucellosis	Brucellosis	Brucellosis	Brucellosis	Brucellosis	Brucellosis	Brucellosis	Brucellosis
84	Syphilis	Syphilis	Syphilis	Syphilis	Syphilis	Syphilis	Syphilis	Syphilis	Syphilis	Brucellosis	Brucellosis	Brucellosis	Brucellosis	Brucellosis	Brucellosis	Brucellosis	Brucellosis	Brucellosis
85	Syphilis	Syphilis	Syphilis	Syphilis	Syphilis	Syphilis	Syphilis	Syphilis	Syphilis	Brucellosis	Brucellosis	Brucellosis	Brucellosis	Brucellosis	Brucellosis	Brucellosis	Brucellosis	Brucellosis
86	Syphilis	Syphilis	Syphilis	Syphilis	Syphilis	Syphilis	Syphilis	Syphilis	Syphilis	Brucellosis	Brucellosis	Brucellosis	HFRS	Brucellosis	Brucellosis	Brucellosis	Brucellosis	Brucellosis
87	Syphilis	Syphilis	Syphilis	Syphilis	Syphilis	Syphilis	Syphilis	Syphilis	Syphilis	Brucellosis	Brucellosis	HFRS	Brucellosis	HFRS	Brucellosis	Brucellosis	Brucellosis	Brucellosis
88	Syphilis	Syphilis	Syphilis	Syphilis	Syphilis	Syphilis	Syphilis	Syphilis	Syphilis	Brucellosis	Brucellosis	HFRS	HFRS	HFRS	Brucellosis	Brucellosis	Brucellosis	Brucellosis
89	Syphilis	Syphilis	Syphilis	Syphilis	Syphilis	Syphilis	Syphilis	Syphilis	Syphilis	Brucellosis	Brucellosis	Brucellosis	HFRS	HFRS	Brucellosis	Brucellosis	Brucellosis	Brucellosis
90	Syphilis	Syphilis	Syphilis	Syphilis	Syphilis	Syphilis	Syphilis	Syphilis	Syphilis	Brucellosis	Rabies	Brucellosis	Brucellosis	Brucellosis	HFRS	Brucellosis	Brucellosis	Brucellosis
91	Syphilis	Syphilis	Syphilis	Syphilis	Syphilis	Syphilis	Syphilis	Syphilis	Syphilis	Brucellosis	HFRS	Brucellosis	Brucellosis	Brucellosis	Brucellosis	Brucellosis	HFRS	Brucellosis
92	Syphilis	Syphilis	Syphilis	Syphilis	Syphilis	Syphilis	Syphilis	Syphilis	Syphilis	Brucellosis	Brucellosis	Rabies	HFRS	Brucellosis	HFRS	HFRS	Brucellosis	Brucellosis
93	Syphilis	Syphilis	Syphilis	Syphilis	Syphilis	Syphilis	Syphilis	Syphilis	Syphilis	Brucellosis	Brucellosis	HFRS	Brucellosis	HFRS	Brucellosis	HFRS	Brucellosis	Brucellosis
94	Syphilis	Syphilis	Syphilis	Syphilis	Syphilis	Syphilis	Syphilis	Syphilis	Syphilis	Brucellosis	Brucellosis	HFRS	Brucellosis	Hydatid disease	HFRS	Brucellosis	Brucellosis	Brucellosis
95	Syphilis	Syphilis	Syphilis	Syphilis	Syphilis	Syphilis	Syphilis	Syphilis	Syphilis	Brucellosis	Brucellosis	HFRS	Brucellosis	HFRS	Brucellosis	HFRS	Brucellosis	Brucellosis
96	Syphilis	Syphilis	Syphilis	Syphilis	Syphilis	Syphilis	Syphilis	Syphilis	Syphilis	Brucellosis				HFRS	HFRS	HFRS	Brucellosis	
97	Syphilis	Syphilis	Syphilis	Syphilis	Syphilis	Syphilis	Syphilis	Syphilis	Syphilis	Brucellosis			Brucellosis	HFRS	Brucellosis	HFRS	Brucellosis	
98	Syphilis	Syphilis	Syphilis	Syphilis	Syphilis	Syphilis	Syphilis	Syphilis	Syphilis	Brucellosis			Brucellosis	Brucellosis	Brucellosis		Brucellosis	
99	Syphilis	Syphilis	Syphilis	Syphilis	Syphilis	Syphilis	Syphilis	Syphilis	Syphilis	Brucellosis		Rabies		Brucellosis	HFRS			
≥100	Syphilis	Syphilis	Syphilis	Syphilis	Syphilis	Syphilis	Syphilis	Syphilis	Syphilis	Brucellosis	HFRS	Brucellosis			Brucellosis	Brucellosis Hydatid disease		Brucellosis

Figure S9. Changing trends in the age-specific incidences of other six NIDs among the older people, stratified by sex, as analyzed using Join-Point regression model.

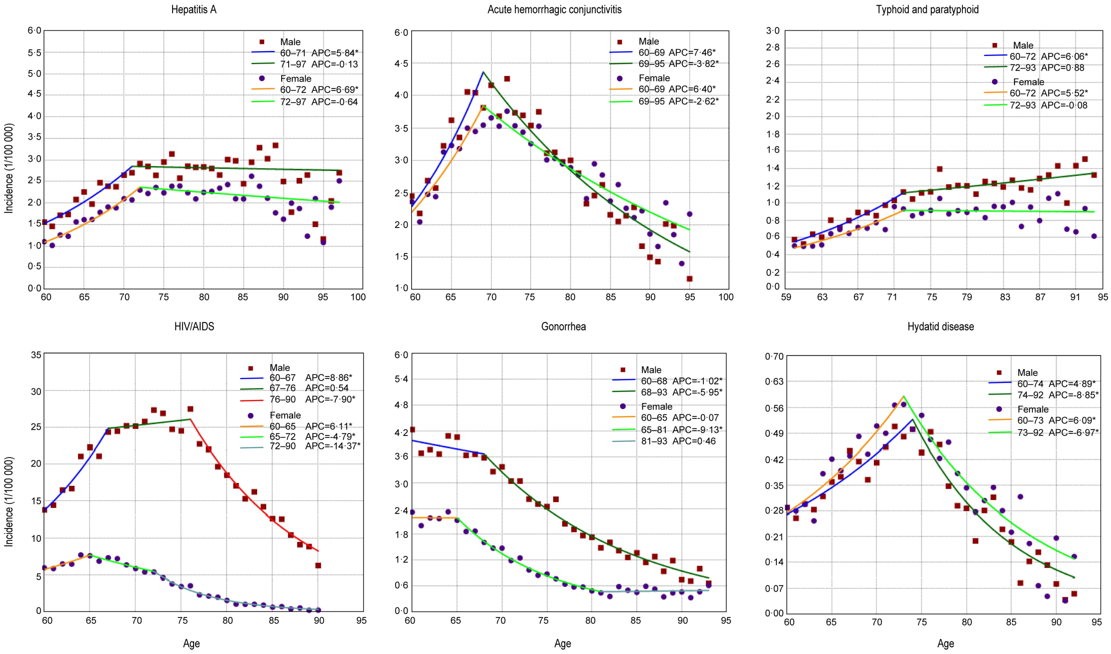

Figure S10. Changing trends in the age-specific incidences of infectious diseases among the older people, stratified by urban areas, as analyzed using Join-Point regression model. a. Respiratory diseases. b. Gastrointestinal or enteroviral diseases. c. Sexually transmitted or bloodborne diseases. d. Vector-borne or zoonotic diseases. HFRS: hemorrhagic fever with renal syndrome. Note: The significant annual percent changes (APCs) at two-sided P<0.05 are marked by "*".

a

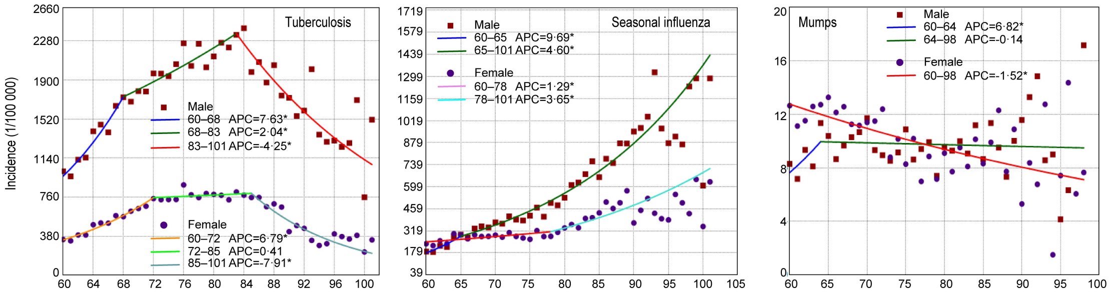

b

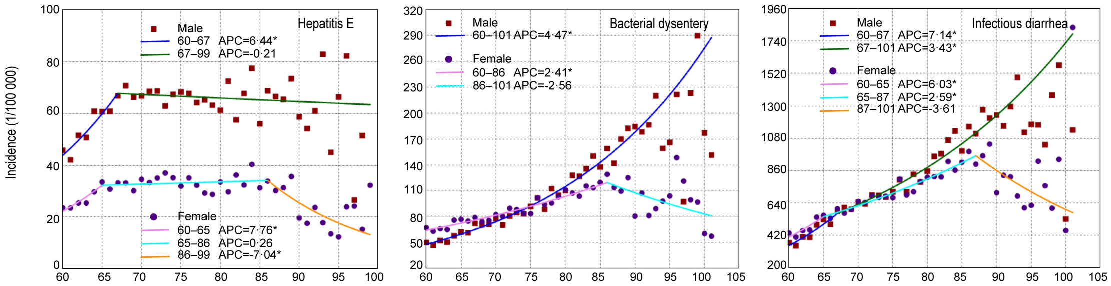

c

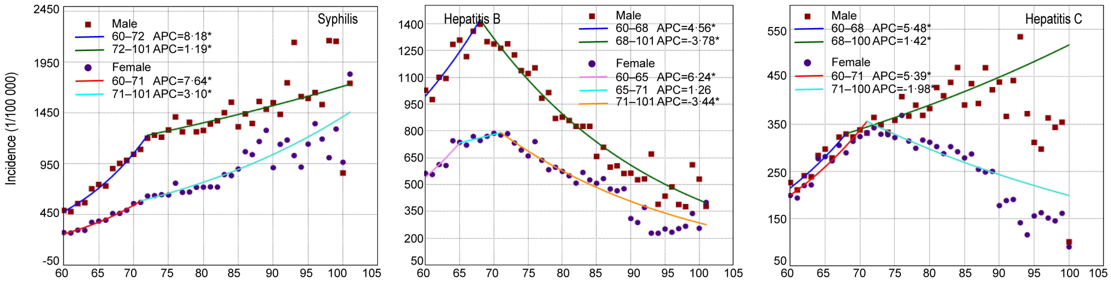

d

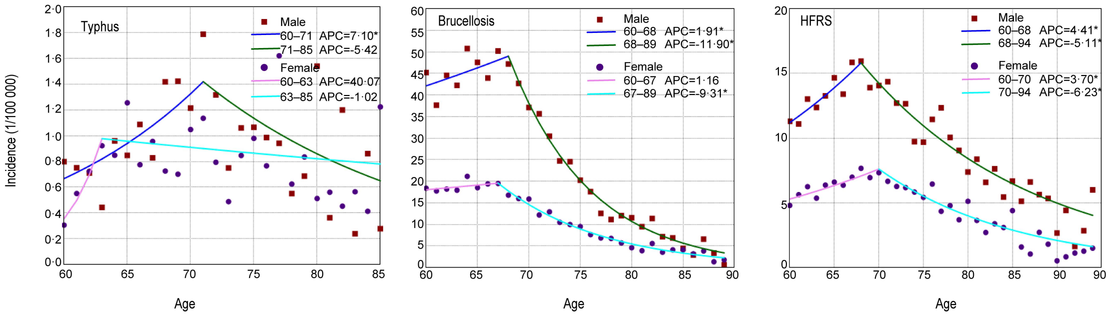

Figure S10. Continued.

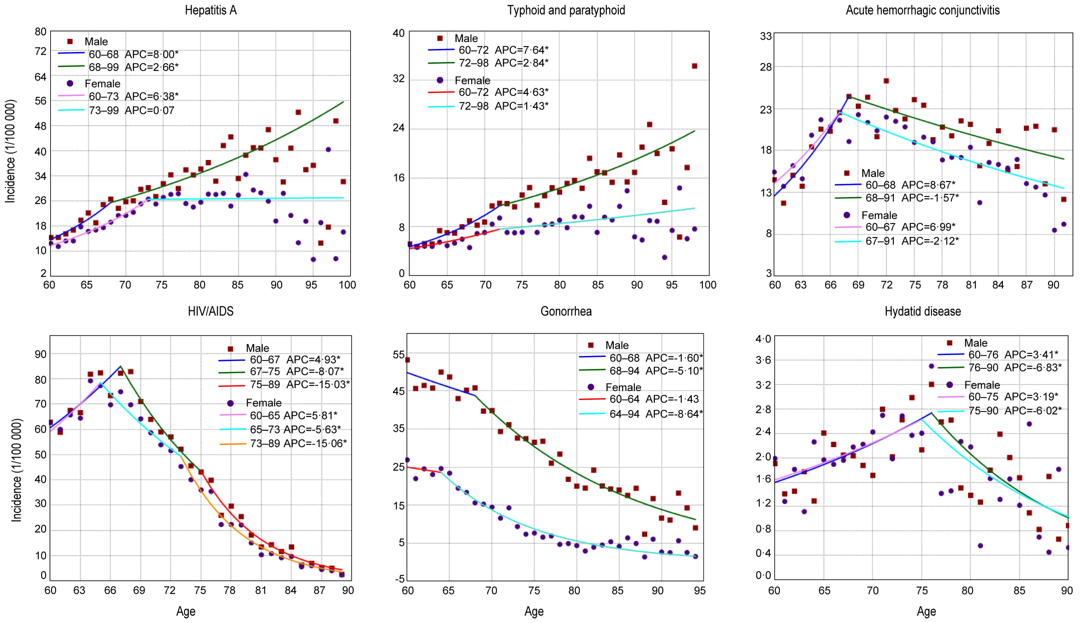

Figure S11. Changing trends in the age-specific incidences of infectious diseases among the older people, stratified by rural areas, as analyzed using Join-Point regression model. a. Respiratory diseases. b. Gastrointestinal or enteroviral diseases. c. Sexually transmitted or bloodborne diseases. d. Vector-borne or zoonotic diseases. HFRS: hemorrhagic fever with renal syndrome. Note: The significant annual percent changes (APCs) at two-sided P<0.05 are marked by "*".

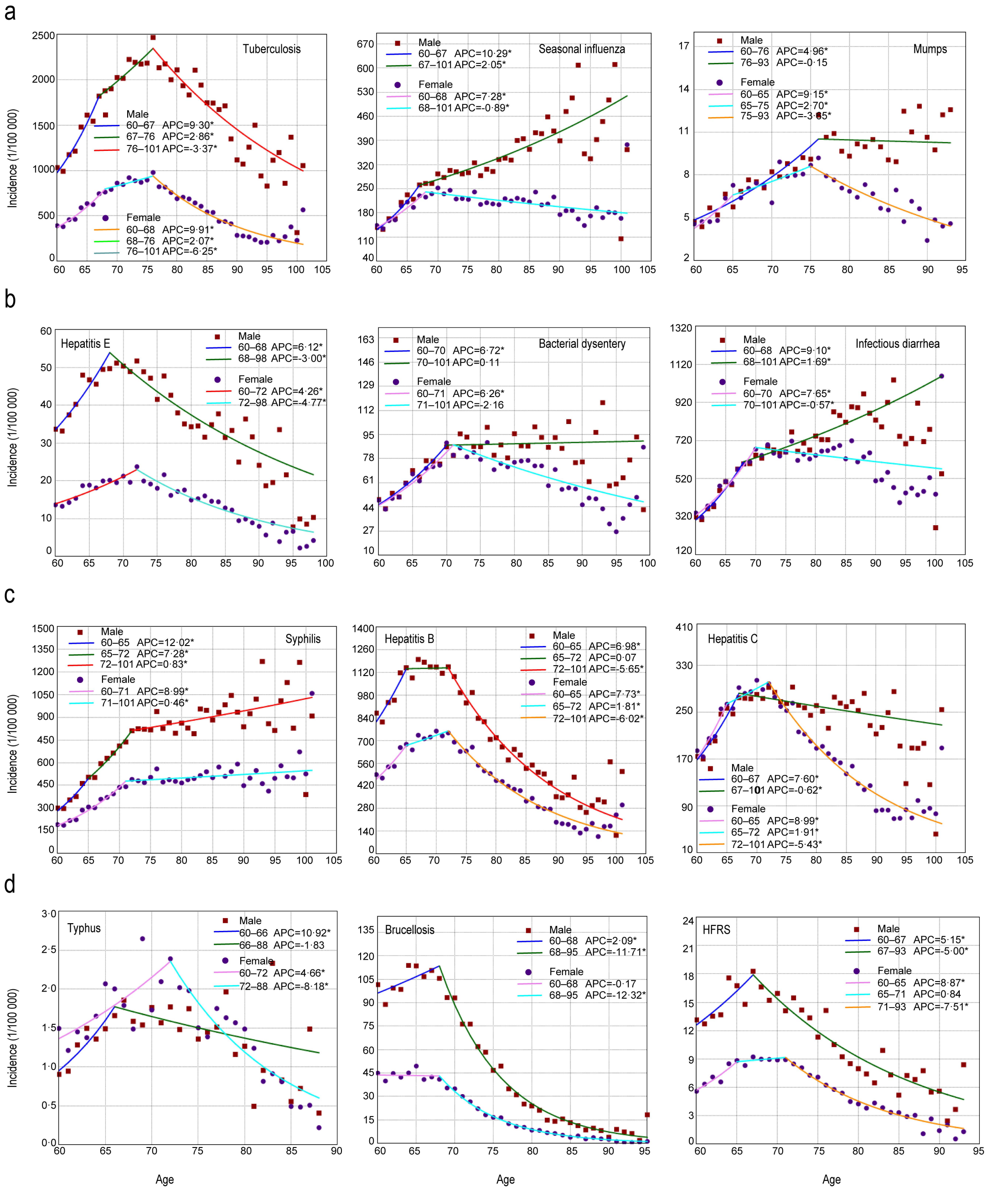

Figure S11. Continued.

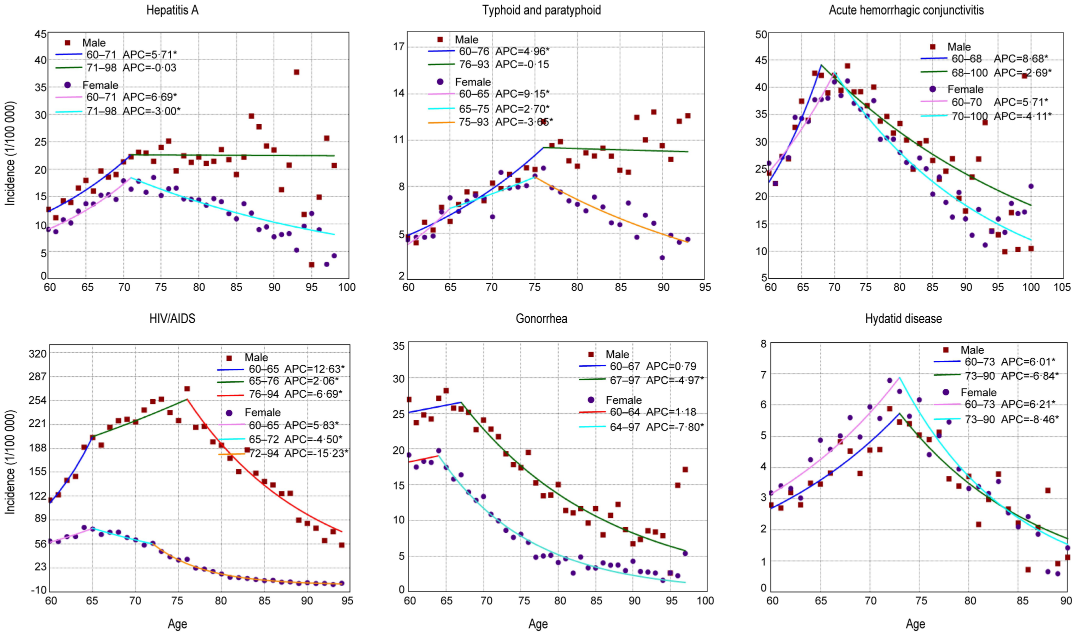

Figure S12. Changing trends in the age-specific incidences of infectious diseases among the older people, stratified by year, as analyzed using Join-Point regression model. a. Respiratory diseases. b. Gastrointestinal or enteroviral diseases. c. Sexually transmitted or bloodborne diseases. d. Vector-borne or zoonotic diseases. HFRS: hemorrhagic fever with renal syndrome. Note: The significant annual percent changes (APCs) at two-sided P<0.05 are marked by "*".

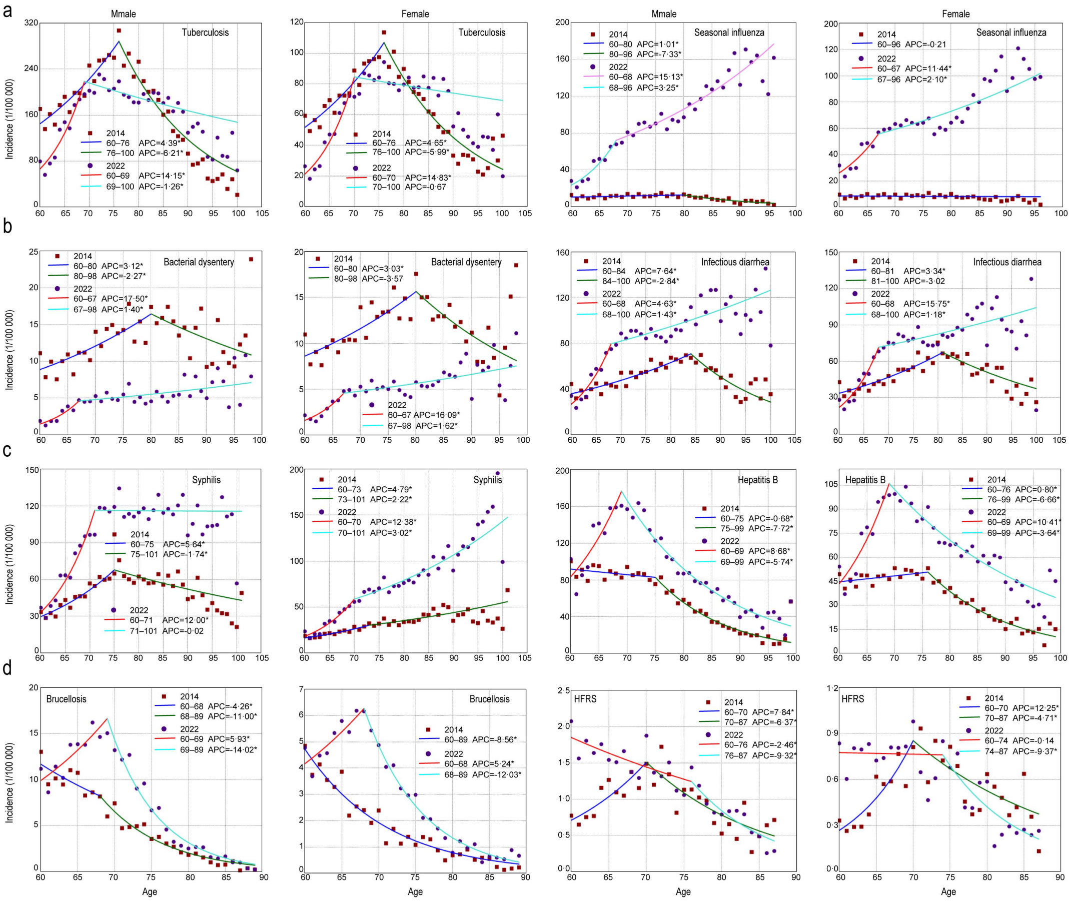

Figure S12. Continued. AHC: acute hemorrhagic conjunctivitis.

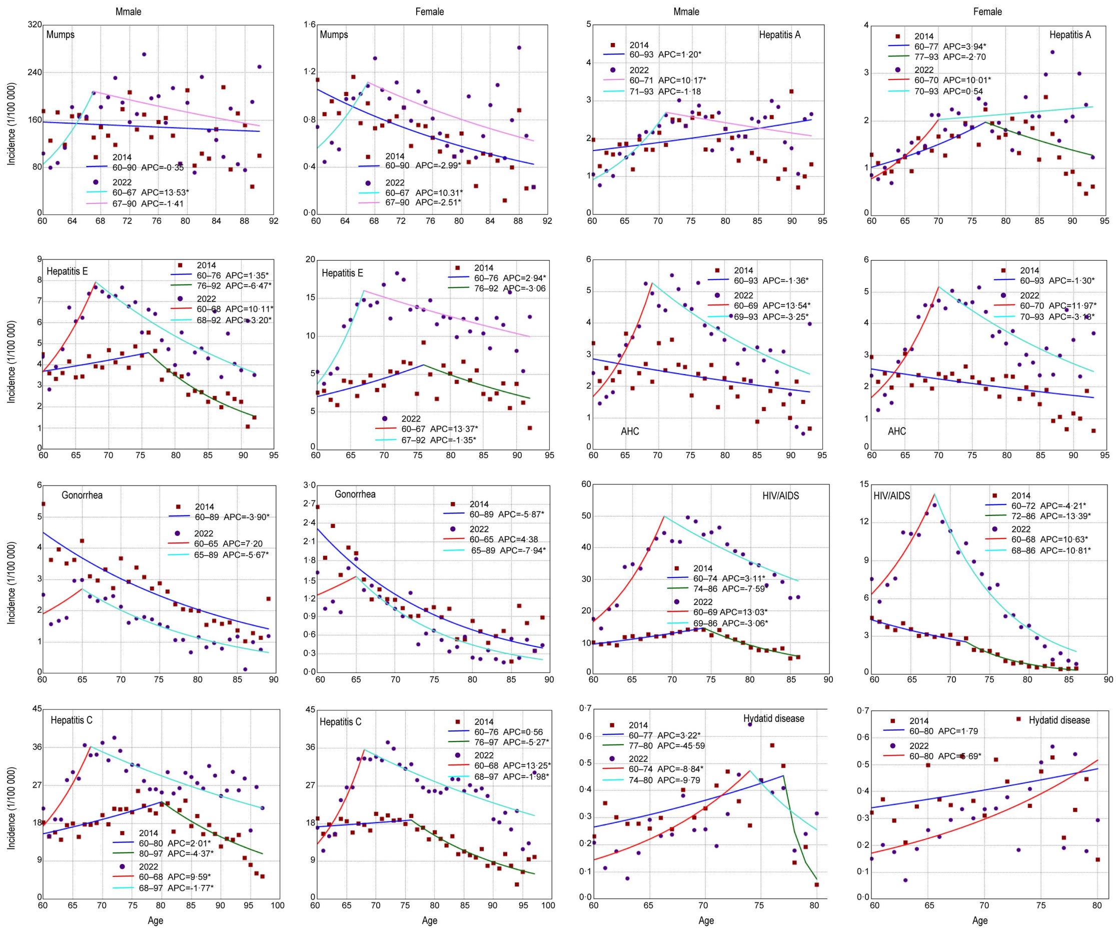

Supplement: Supplementary Appendix [file mmc1.pdf]
