# Supplementary material for: Epidemiological analysis of infectious diseases in older people in China from 2014 to 2022: a population-based study
Source: Lancet Reg Health West Pac. 2025 Nov 3;64:101729. doi: 10.1016/j.lanwpc.2025.101729 (PMC12677096; doi:10.1016/j.lanwpc.2025.101729)
Supplement: Translated Abstract-Fang [file mmc2.docx]

**摘要**

**背景**：全世界正在经历前所未有的快速人口老龄化，随之而来的老年人群传染病流行将对国家公共卫生构成严峻挑战。

**方法**：我们从中国传染病监测与控制项目（CISDCP）中提取了2014年至2022年中国大陆地区60岁及以上人群的21种法定传染病个案数据，全面分析了这些传染病在老年人群中的流行病学特征，并评估了年龄、年份以及COVID-19大流行对这些疾病的影响

**结果**：研究期间，共报告了8,604,064例21种老年人群主要传染病，总年发病率为362.10/10万。性传播或血液传播疾病（占比45.23%）是最常见的疾病类型。值得注意的是，梅毒的排名从第四位上升至第三位，而HIV/AIDS从第八位上升至第七位；呼吸道疾病（占比34.88%）呈持续下降趋势。尽管肺结核的发病率每年下降6.4%（年平均变化，P<0.001），但肺结核仍然是最常见的呼吸道疾病；除其他感染性腹泻病和戊型肝炎外，其他胃肠道或肠道病毒疾病（18.35%）均保持下降态势；病媒传播或人畜共患疾病（1.54%）的发病率一直保持在较低水平，但布鲁氏菌病在COVID-19期间有所增加。拐点分析显示，肺结核、其他感染性腹泻病、乙型肝炎等13种疾病的特定年龄发病率随着年龄增长呈现二次分布（倒“V”形）。不同地区之间存在显著差异，蒙新区和华南区的老年人群仍承担着较高的传染病负担。

**解释**：尽管中国在老年人群传染病防控方面取得了显著进展，但对于一些高发疾病，如肺结核、乙型肝炎、其他感染性腹泻病等，仍需制定并实施更具针对性的防控策略，尤其是在高发地区和关键年龄段。

**资助**：中国国家重大研发计划。

This translation in Chinese was submitted by the authors and we reproduce it as supplied. It has not been peer reviewed. Our editorial processes have only been applied to the original abstract in English, which should serve as reference for this manuscript
